# Calcium-activated chloride channel regulator 1 (CLCA1) forms non-covalent oligomers in colonic mucus and has mucin 2–processing properties

**DOI:** 10.1074/jbc.RA119.009940

**Published:** 2019-09-29

**Authors:** Elisabeth E. L. Nyström, Liisa Arike, Erik Ehrencrona, Gunnar C. Hansson, Malin E. V. Johansson

**Affiliations:** Department of Medical Biochemistry and Cell Biology, Institute of Biomedicine, University of Gothenburg, SE-405 30 Gothenburg, Sweden

**Keywords:** mucin, intestine, colitis, protease, metalloprotease, gastrointestinal tract, Gob-5, mouse Clca3, mucin-2 (MUC2), von Willebrand type D domain (VWD)

## Abstract

Calcium-activated chloride channel regulator 1 (CLCA1) is one of the major nonmucin proteins found in intestinal mucus. It is part of a larger family of CLCA proteins that share highly conserved features and domain architectures. The CLCA domain arrangement is similar to proteins belonging to the ADAM (a disintegrin and metalloproteinase) family, known to process extracellular matrix proteins. Therefore, CLCA1 is an interesting candidate in the search for proteases that process intestinal mucus. Here, we investigated CLCA1's biochemical properties both *in vitro* and in mucus from mouse and human colon biopsy samples. Using immunoblotting with CLCA1-specific antibodies and recombinant proteins, we observed that the CLCA1 C-terminal self-cleavage product forms a disulfide-linked dimer that noncovalently interacts with the N-terminal part of CLCA1, which further interacts to form oligomers. We also characterized a second, more catalytically active, N-terminal product of CLCA1, encompassing the catalytic domain together with its von Willebrand domain type A (VWA). This fragment was unstable but could be identified in freshly prepared mucus. Furthermore, we found that CLCA1 can cleave the N-terminal part of the mucus structural component MUC2. We propose that CLCA1 regulates the structural arrangement of the mucus and thereby takes part in the regulation of mucus processing.

## Introduction

2018 marked the 20^th^ anniversary of the discovery of calcium-activated chloride channel regulator 1 (CLCA1),^2^ and despite intense research its molecular and physiological functions remain largely unknown (1, 2). Most current research focuses on the function of CLCA1 in the airways where it is normally virtually absent but is up-regulated in diseases such as asthma and COPD (3, 4). However, CLCA1 is primarily expressed along the gastrointestinal tract under normal conditions and is highly abundant in the intestinal mucus layer (5).

**Figure 1.**
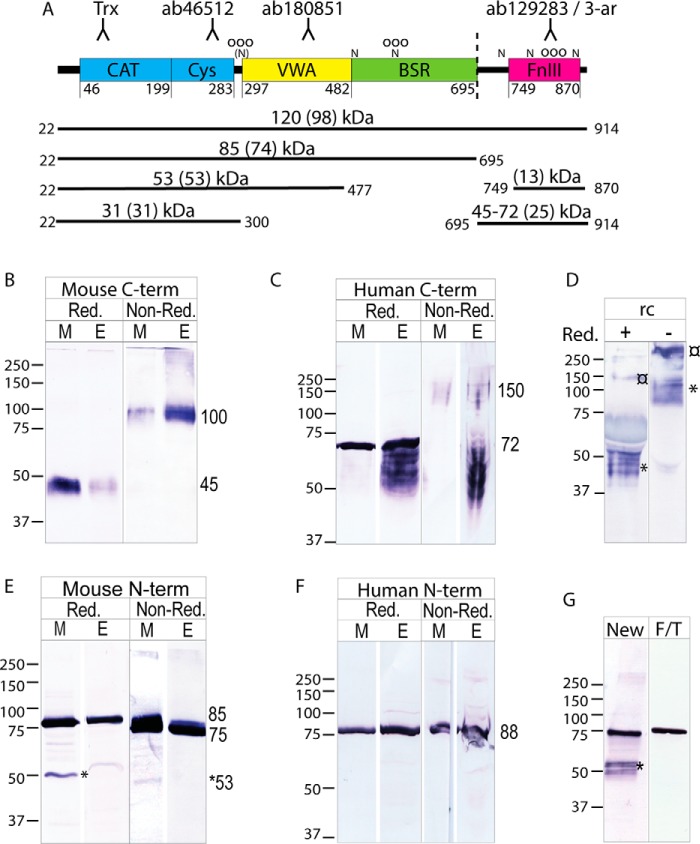
**CLCA1 in colonic mucus and epithelium.** *A*, schematic outline of CLCA1's domain structure with domain boundaries based on human CLCA1 sequence (adapted from Ref. 12) and antibody epitopes. As CLCA1 is secreted, the 21-amino-acid signal sequence is not included. *Horizontal lines* represent full-length and truncated CLCA1 proteins with start and end amino acid positions (in its secreted form). The observed molecular mass is given together with the theoretical molecular mass (in *parentheses*). *Dashed line*, cleavage site; *N*, predicted *N*-glycosylation site; *(N)*, predicted N-glycosylation site with proline in X1 position; *O*, predicted *O*-glycosylation site. *B* and *C*, Western blotting of mucus (*M*) and epithelial lysate (*E*) from mouse distal colon (*B*) or human sigmoid colon (*C*) analyzed under reduced (*Red.*) and nonreduced (*Non-Red.*) conditions detected with C-terminal CLCA1–recognizing antibodies (mouse, 3-ar; human, ab129283). *D*, supernatant from rCLCA1-expressing CHO-K1 cells analyzed under reducing (+) or nonreducing (−) conditions blotted with a C terminus (3-ar)–recognizing antibody. ¤ denotes uncleaved CLCA1. * denotes cleaved C-terminal CLCA1. *E* and *F*, Western blot analysis of mouse and human colonic material (*E* and *F*, respectively) under reducing (*Red.*) and nonreducing (*Non-Red.*) conditions blotted with N-terminal CLCA1–recognizing antibodies (mouse, ab46512; human, Trx-CLCA1). Both mucus (*M*) and epithelial lysates (*E*) were analyzed. *G*, immunoblot using the N terminus–recognizing antibody ab46512 on a mouse colonic mucus sample analyzed fresh (*New*) or after freeze/thawing (*F/T*). * denotes a 53-kDa band. Molecular mass references are given in kDa. *Numbers* to the *right* of the blots denote the determined molecular mass (in kDa) of the main bands in the blot.

Murine Clca1 has previously been named Gob-5 and mClca3 but was recently renamed according to the human CLCA nomenclature (6). CLCA1 belongs to a family of CLCAs with four human (hCLCA1–4) and eight murine homologs (mClca1, -2, -3a, -3b, -3c, -4a, -4b, and -4c) of which only CLCA1/Clca1 is secreted (6). The CLCAs share common features and highly conserved protein domains (Fig. 1*A*). An N-terminal signal sequence directs the proteins to the secretory pathway, and all CLCAs undergo intracellular autocatalytic self-cleavage at a conserved site to produce a larger N-terminal and a smaller C-terminal product (7–10). The resulting N-terminal product harbors a zinc-dependent metalloprotease catalytic domain (CAT) with a conserved HE*XX*E motif in conjunction with a cysteine-rich region (Cys) (8, 11). A von Willebrand domain type A (VWA) domain with a conserved metal ion–dependent adhesion site (MIDAS) followed by an unassigned β-sheet–rich (BSR) domain is also found in the N terminus. A fibronectin type III (FnIII) domain is suggested in the C terminus (12).

This domain structure resembles that of “a disintegrin and metalloproteinase” (ADAM) proteins, although CLCA1 lacks a propeptide and a disintegrin domain (8). Several ADAMS are known to cleave extracellular matrix proteins, *e.g.* collagen (13). However, currently the only known substrate for CLCA1 is itself (8). CLCA1 has the potential for protein–protein interactions with other mucus components mediated by either the VWA or FnIII domain, but no such interactions have yet been described. However, it is suggested that the VWA domain confers MIDAS-dependent interaction between CLCA1 and the ion channel TMEM16A *in vitro*, but the intestinal physiological implication of such an interaction remains unclear (12).

CLCA1 was initially believed to form a calcium-activated chloride channel based on altered ion currents in cells overexpressing CLCA1 and an initial erroneous prediction of transmembrane domains (1, 2). However, the secreted nature of CLCA1 contradicts this, and it is instead assumed to be an ion channel regulator or accessory protein (8, 14–16). Conversely, we recently found that CLCA1, independently of ion channel involvement, acts as a metalloprotease in the intestinal mucus and alters mucus dynamics and structure, thus providing an alternative function for CLCA1 (17). However, *Clca1*^−/−^ animals did not exhibit a strong intestinal mucus phenotype in terms of mucus dynamics and penetrability due to compensatory protease activity.

Intestinal mucus protects the underlying epithelium by lubrication and by limiting contact between the epithelium and the gut-residing bacteria. This limitation is especially important in the distal colon where the bacterial burden is the highest (18). Disruption of the mucus structure is correlated with colitis both in humans and mice, whereas intestinal mucus stagnation is observed during cystic fibrosis (19, 20). Thus, understanding factors that control mucus structure and processing may provide useful insight to the pathology of mucus-associated diseases such as ulcerative colitis and cystic fibrosis.

MUC2 provides the structural skeleton of intestinal mucus. It is a large (>5000-amino-acid) gel-forming mucin with the following domain structure that is conserved between species: von Willebrand D assemblies D1, D2, D′, and D3; first cysteine domain (CysD); small proline, threonine, and serine–rich (PTS) domain; second CysD; large PTS domain; C-terminal D4 followed by von Willebrand C domain assemblies; and a cysteine-knot (CK) domain. The von Willebrand D assemblies can be further subdivided into von Willebrand D (VWD) domain, C8 module, trypsin inhibitor–like (TIL) domain, and a fibronectin type I–like (E)-domain, except for D′, which only contains a TIL and E-domain (21). The two PTS domains are heavily *O*-glycosylated during biosynthesis, generating a mature MUC2 molecule with a molecular mass in the MDa range. Furthermore, MUC2 forms very large oligomers by disulfide-mediated C-terminal dimerization of the CK domain and N-terminal trimerization of the D3 assembly (22, 23). The oligomers are tightly packed in intracellular secretory granules but unfold into large netlike sheets after secretion (24). The interactions between MUC2 sheets are less well-defined but might be mediated by noncovalent interactions between D3 assemblies or covalent isopeptide bonds between CysD domains generated by transglutaminases (25, 26). In the closely related protein von Willebrand factor (VWF), D1–D2 form a propeptide that is cleaved off but remains associated with the mature VWFs and stabilizes its intracellular assembly (27). A similar role for the MUC2 D1–D2 has been suggested (24).

Most of the available biochemical information concerning CLCA1 is derived from *in vitro* experiments. We therefore investigated the biochemical properties of CLCA1 *in vivo* in colonic epithelium and mucus under reducing, nonreducing, and native conditions to characterize the processing and features of intestinal CLCA1. Furthermore, as we have previously observed that CLCA1 has mucus-modulating properties (17), we tested the hypothesis that MUC2 serves as a substrate for CLCA1. Our results indicate that a novel N-terminal cleavage product of CLCA1 encompassing the CAT/Cys and VWA domains is present in colonic mucus and is able to process the N terminus of MUC2. The suggested cleavage of MUC2 provides a mechanism describing how CLCA1 could alter intestinal mucus structure.

## Results

### CLCA1 in colonic mucus and epithelium

To better understand how CLCA1 is processed in the colon, mucus and epithelial lysates from mouse and human sigmoid colon were analyzed by Western blotting using CLCA1-specific antibodies. Despite being previously reported as unstable, the monomeric C-terminal cleavage product of CLCA1 was detected in mucus and epithelium from both mouse and human samples under reducing conditions at 45 and 72 kDa in mouse and human samples, respectively (Fig. 1, *B* and *C*, *Red.*) (14). To investigate whether the bands at 50–72 kDa detected in human epithelial samples (Fig. 1*C*, “*E*”) might correspond to immaturely glycosylated C terminus, recombinant CLCA1 (rCLCA1) was treated with peptide:*N*-glycosidase F (PNGaseF), sialidases, and *O*-glycosidase to remove *N*-glycans and GalGalNAc-glycans, respectively. PNGaseF treatment caused a noticeable size shift of the C terminus, indicating that C-terminal CLCA1 is *N*-glycosylated (Fig. S1*A*). Further treatment with sialidase and *O*-glycosidase shifted the bands down. The PNA lectin detected both N- and C-terminal rCLCA1, confirming glycosylation of both termini (Fig. S1*B*). The C terminus formed a single band when deglycosylated (Fig. S1*A*). The smaller C-terminal bands detected in human lysate thus likely belong to immaturely glycosylated C terminus. Conversely, only a single band at 72 kDa corresponding to the maturely glycosylated C terminus was detected in reduced human mucus samples (Fig. 1*C*, *Red.*, “*M*”). Under nonreducing conditions, the mature C terminus appeared at a size corresponding to a C-terminal dimer in both mouse and human mucus and epithelium (100 and 150 kDa in mouse and human samples, respectively) (Fig. 1, *B* and *C*, *Non-Red.*). Dimerization appeared to occur intracellularly as the dimer was detected in epithelial samples. However, the immature C-terminal material in human epithelial lysate did not appear as dimers, indicating that dimerization takes place after full glycosylation of the C-terminal. Furthermore, traces of larger C-terminal complexes were detected in epithelial lysates from both mouse and human, which may be aggregates of immaturely processed C-terminal. However, the nature of these was not investigated further. A C-terminal dimer was also detected under nonreducing conditions when analyzing supernatant from hCLCA1-transfected CHO-K1 cells, indicating that this was a C-terminal homodimer rather than a heterodimeric complex with another mucus protein (Fig. 1*D*).

The monomeric N-terminal part of CLCA1 was readily detected at 85 (mouse) or 88 kDa (human) in both mucus and epithelium from both species under reducing conditions (Fig. 1, *E* and *F*, *Red.*). In addition, a smaller fragment at 53 kDa was identified in mouse mucus samples by N-terminal antibodies, which was lost after freezing and thawing of the sample (Fig. 1, *E* and *G*, marked with *). A corresponding band was occasionally also detected in fresh human samples (see Fig. 3*A*). The N terminus remained monomeric under nonreducing conditions (Fig. 1, *E* and *F*, *Non-Red.*). The small size shift seen in mouse material might be due to intracellular disulfide bonds that slightly alter the migration pattern of the N terminus.

PNGaseF treatment of rCLCA1 induced a size shift of the N terminus, indicating that the predicted *N*-glycosylation sites are indeed glycosylated (Fig. S1*C*). *O*-Glycosidase treatment did not induce a noticeable size shift, but the N-terminal was detected by PNA lectin, possibly indicating *O*-glycosylation.

Uncleaved CLCA1 was not detected in mucus samples and barely, if at all, in epithelial samples, indicating that the vast majority of CLCA1 is cleaved intracellularly. However, some uncleaved rCLCA1 could be detected in supernatant from transfected CHO-K1 cells (Fig. 1*D*, marked with ¤). It thus appears that cleavage is not required for secretion of CLCA1. Likewise, it does not seem crucial for dimerization as the secreted uncleaved material was found as a dimer under nonreducing conditions.

### CLCA1 oligomerization

**Figure 2.**
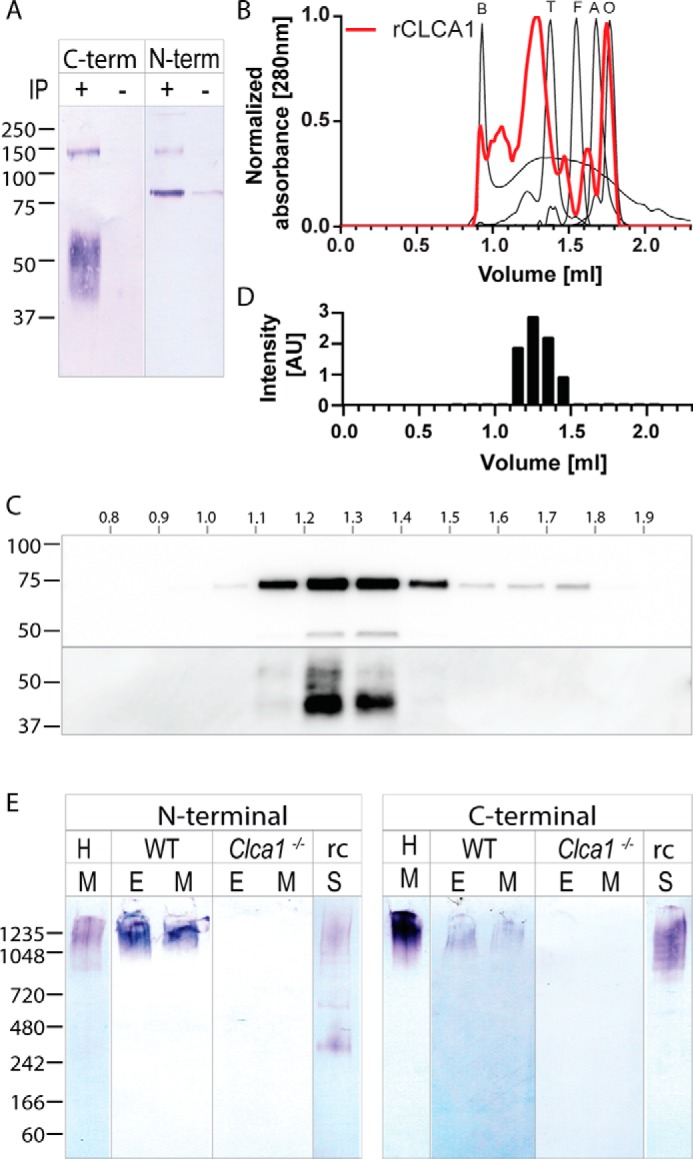
**CLCA1 oligomerization.** *A*, detection of C-terminal and N-terminal rCLCA1 in the eluate after Dynabeads His pulldown of C-terminally tagged CLCA1. +, rCLCA1-His pulldown; −, eluate from pulldown on supernatant from mock-transfected cells. *B*, size-exclusion elution profiles of molecular size standards (blue dextran (B), 2000 kDa; thyroglobulin (*T*), 670 kDa; ferritin (*F*), 440 kDa; aldolase (*A*), 158 kDa; ovalbumin (*O*), 44 kDa) and rCLCA1-His from Superose 6 Increase 3.2/300. *C*, detection of N- and C-terminal (*upper* and *lower panels*, respectively) rCLCA1 in elution fractions from *B. D*, intensity profile of bands from *C*, *top panel*, aligned to the corresponding elution fractions in *B. E*, native PAGE of mucus (*M*) from human sigmoid colon (*H*), mucus and epithelial lysate (*E*) from WT and *Clca1*^−/−^ mouse colon, and spent supernatant (*S*) from rCLCA1-expressing CHO-K1 cells (*rc*) blotted and probed with either an N-terminal (*left panel*) or C-terminal (*right panel*) CLCA1–recognizing antibody. Antibodies used are the same as those in Fig. 1, *B*, *C*, *E*, and *F*. Molecular mass references are given in kDa. *IP*, immunoprecipitation; *AU*, arbitrary units.

Association between N- and C-terminal CLCA1 was analyzed by a pulldown assay. After pulldown of C-terminally His_6_-tagged rCLCA1, both the C- and the N-terminal cleavage products could be detected in the eluate (Fig. 2*A*), thus indicating that the cleavage products remain noncovalently associated. Furthermore, rCLCA1 N and C termini eluted together between blue dextran (2000 kDa) and thyroglobulin (669 kDa) in size-exclusion gel chromatography, indicating that CLCA1 is able to form oligomers (Fig. 2, *B–D*). However, as rCLCA1 eluted outside the linear range of the standards, we were not able to determine the precise molecular mass of the oligomers by this method. To confirm the oligomeric nature of CLCA1 *in vivo*, mucus and epithelial lysates from human sigmoid colon or WT and *Clca1*^−/−^ mice as well as rCLCA1 were analyzed by native PAGE. After Western blotting and analysis with either N or C terminus–recognizing antibodies, CLCA1 was found in large complexes with an apparent mass of >1 MDa in all human and WT mouse samples and using rCLCA1 (Fig. 2*E*). Thus, CLCA1 seems to form octamers by noncovalent bonds between N termini besides the interaction between N and C termini.

### Domains of CLCA1

**Figure 3.**
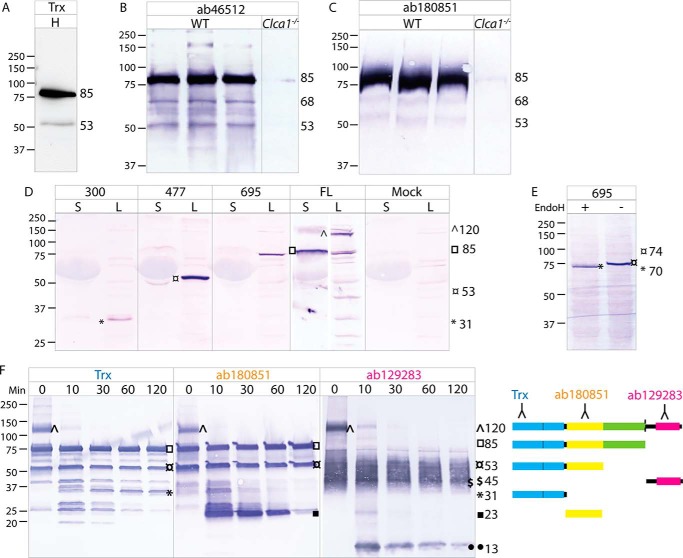
**Domains of CLCA1.** *A*, immunoblot of human sigmoid colon mucus (*H*) with the Trx-hCLCA1 antibody against the CAT/Cys domain. *B* and *C*, colonic mucus from WT and *Clca1*^−/−^ mice immunoblotted with an antibody directed against the CAT/Cys (ab46512) (*B*) or VWA domain (ab180851) (*C*). *D*, detection of N-terminal truncated (300, 477, and 695) and full-length (*FL*) rCLCA1 in spent supernatant (*S*) and lysate (*L*) from transfected CHO-K1 cells. Spent supernatant and lysate from mock-transfected cells were used as controls. *E*, Western blotting of EndoH-treated (+) and control (−) lysate from cells expressing the 1–695 truncated protein probed with N terminus–recognizing antibody Trx-CLCA1. *F*, immunoblots of rCLCA1 after limited tryptic proteolysis with samples at *t* = 0, 10, 30, 60, and 120 min with antibodies directed against the very N-terminal part of CLCA1 (Trx-hCLCA1), VWA domain (ab180851), or C-terminal CLCA1 (ab129283). A schematic representation of the suggested main products is shown to the *right. Blue*, CAT/Cys; *yellow*, VWA; *green*, BSR; *pink*, FnIII. Molecular mass references are given in kDa. *Numbers* to the *right* of the blots denote the determined molecular mass (in kDa) of the main bands (marked by *symbols*) in the blot.

To characterize the 53-kDa N-terminal product of CLCA1 occasionally observed in mucus samples, fresh mucus from both human, WT mouse, and *Clca1*^−/−^ mouse colon was analyzed by SDS-PAGE and immunoblotted with antibodies directed against different N-terminal epitopes. The 53-kDa band was specifically detected with antibodies against both the CAT/Cys domain and VWA domain (Fig. 3, *A–C*). In addition, a 68-kDa band could be observed in WT mouse mucus (Fig. 3, *B* and *C*).

Furthermore, truncated rCLCA1 encompassing the CAT/Cys (aa 1–300), CAT/Cys + VWA (aa 1–477), or the full N terminus (aa 1–695) was expressed in CHO-K1 cells (Fig. 3*D*). The truncated CLCA1 proteins were detected at 31, 53, and 85 kDa, respectively. For comparison, an 85-kDa N-terminal product as well as some uncleaved material at 120 kDa could be detected from cells expressing the full-length CLCA1. The molecular mass of the CAT/Cys + VWA (477) protein was similar to the mass of the 53-kDa product detected in mucus. As the 53-kDa product in mucus was detected with antibodies against CAT/Cys and VWA, this strongly suggests that both domains comprise part of a novel N-terminal product of CLCA1.

Discrepancy between the theoretical and observed sizes of the full N-terminal fragment, but not the smaller truncated proteins, indicates post-translational modifications of the BSR domain (Figs. 1*A* and 3*D*). These are possibly glycans as two *N*-glycosylation sites are predicted in this region. Similarly, the observed molecular mass of secreted C-terminal CLCA1 is considerably larger than the theoretical molecular mass (Figs. 1*A* and 3*F*). As for the BSR domain, several *N*-glycosylation sites are predicted in addition to a small stretch of amino acids with several prolines, threonines, and serines (aa 863–881), which indicate possible *O*-glycosylation of the C terminus. Glycosylation of CLCA1 is supported by size shifts upon deglycosylation and PNA lectin staining (Fig. S1).

Noticeably, only minute levels of the truncated CLCA1 proteins could be detected in the supernatant from the cells in contrast to the full-length protein, which was efficiently secreted (Fig. 3*D*). Endoglycosidase H (EndoH) treatment of cell lysate induced a size shift of the full N-terminal (695) protein, indicating that this material is ER-resident immature N-terminal CLCA1 (Fig. 3*E*). C-terminal CLCA1 thus seems important for proper folding and/or secretion of CLCA1.

Limited tryptic proteolysis of rCLCA1 was used to investigate the domain rigidity of CLCA1. For this, rCLCA1 was treated with trypsin at a 100:1 ratio for 0, 10, 30, 60, and 120 min. The digests were analyzed by SDS-PAGE, Western blotted, and probed with two different N terminus–recognizing antibodies or a C terminus–recognizing antibody (Fig. 3*F*). Some spontaneous degradation of the CLCA1 N terminus was observed in the untreated sample. The uncleaved CLCA1 (120 kDa) detected at *t* = 0 was almost completely absent after 10 min, indicating that the self-cleavage site of CLCA1 is in a scissile part of the protein structure. The 85-kDa full N terminus was still present after 120 min as well as a 53-kDa product that probably encompasses the CAT/Cys + VWA domains as it was detected with antibodies against both. As the intensities of these bands were largely unaffected, these appeared to form relatively stable structures. In addition, a 31-kDa band only detected with the CAT/Cys-recognizing antibody and a 23-kDa product recognized by a VWA-directed antibody indicate that these form discrete domains. The CLCA1 C terminus remained largely intact over the course of the experiment, indicating that it has a protected structure. However, a small fragment at 13 kDa that became fainter over time could be observed. Due to the discrepancy between the theoretical and observed molecular masses of the C-terminal CLCA1, it is not possible to predict the nature of this fragment, although the theoretical size of the FnIII domain is 13 kDa (Fig. 1*A*).

### MUC2 N-terminal cleavage by CLCA1

**Figure 4.**
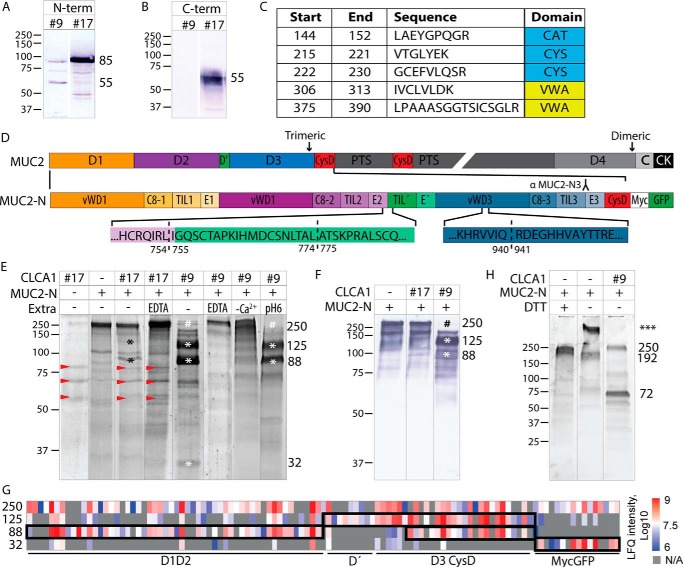
**MUC2 N-terminal cleavage by CLCA1.** *A* and *B*, detection of N- and C-terminal rCLCA1 (*A* and *B*, respectively) in fractions 9 and 17. *C*, identified CLCA1 peptides, with indicated amino acid start and end positions, in the 55-kDa band from fraction 9 in *A* after in-gel tryptic digestion and MS-MS analysis. *D*, schematic outline of MUC2 and MUC2-N with the von Willebrand assemblies D1, D2, D′, D3, D4, CysD, and PTS domains; von Willebrand C assembly), and a CK indicated (adapted from Ref. 25). In MUC2-N, the assembly subdomains and the myc and GFP tags are additionally marked. Epitopes for the MUC2-N3 antibody is *outlined*. The three identified CLCA1 cleavage sites, IRL^754^↓^755^IGQ, TAL^774^↓^775^ATS and VIQ^940^↓^941^RDE, are marked with *dashed lines. E*, *in vitro* cleavage of MUC2-N by rCLCA1, separated by SDS-PAGE and stained with SYPRO Ruby. Different fractions from the rCLCA1 purification were tested (17 and 9) as well as the effect of EDTA addition, removal of Ca^2+^ (−*Ca*^*2*+^), and preincubation of MUC2-N at pH 6. *Red arrows* mark bands from CLCA1 fraction 17, * marks cleavage products, and # marks disappearance of full-length MUC2-N. *F*, immunoblot of *in vitro* cleavage of MUC2-N by rCLCA1 probed with a D3 (MUC2-N3)-recognizing antibody. * marks cleavage products, and # marks disappearance of full-length MUC2-N. *G*, mass spectrometry–based label-free quantification (*LFQ*) of MUC2 peptides in the 250-kDa control and 125-, 88-, and 32-kDa products from rCLCA1-mediated MUC2-N cleavage as shown in *E*. As the product gel bands had overlapping peptides, putative regions of the products are *outlined* based on the peptide label-free quantification intensities and identified new N-terminal sites. *H*, *in vitro* cleavage of MUC2-N by rCLCA1, separated by SDS-PAGE under nonreducing conditions (−*DTT*) and stained with SYPRO Ruby. *** denotes that the molecular mass of the high-molecular-mass band of MUC2-N in nonreducing conditions could not be determined. Molecular mass references are given in kDa. *Numbers* to the *right* of the blots denote the determined molecular mass (in kDa) of the main bands in the blot.

We have previously shown that CLCA1 acts as a metalloprotease in colonic mucus and that proteolytic activity of CLCA1 alters the mucus structure (17). Two purification fractions of rCLCA1 were used to test whether this effect of CLCA1 is mediated by proteolytic processing of MUC2: fraction 17, which mostly contained the 85-kDa N-terminal and 55-kDa C-terminal CLCA1, and fraction 9, which largely lacked these CLCA1 products (Fig. 4, *A* and *B*). Fraction 9 did, however, contain a 55-kDa N-terminal product mapping to the CAT/Cys + VWA domains as evident by Western blotting and MS (Fig. 4, *A* and *C*). Due to the size of full-length MUC2, we used truncated recombinant MUC2 encompassing either the N-terminal part (MUC2-N; Fig. 4*D*) or the C terminus of MUC2 (MUC2-C) in an *in vitro* proteolysis assay with the above-mentioned fractions of CLCA1. No cleavage of MUC2-C could be detected (Fig. S2*A*), whereas an 88-kDa degradation product of MUC2-N was noted after incubation with CLCA1 fraction 17 as well as a trace amount of a 125-kDa product (Fig. 4*E*). CLCA1 fraction 9 resulted in more efficient MUC2-N proteolysis where more or less all intact 250-kDa MUC2-N was cleaved into three fragments with estimated molecular masses of 32, 88, and 125 kDa. This indicated that the suggested N-terminal product of CLCA1 encompassing the CAT/Cys + VWA domains was more proteolytically active than the full N-terminal CLCA1. Addition of the metalloprotease inhibitor EDTA blocked the reactions as did removal of calcium from the assay buffer, indicating that calcium is required for proteolytic activity of CLCA1. Preincubation of MUC2-N at pH 6, however, did not alter CLCA1 activity. To exclude that impurities in the CLCA1 fractions caused the cleavage of MUC2, two additional fractions were tested for MUC2-N proteolysis, and all tested fractions were investigated by proteomics. Neither fraction 7 nor fraction 30 degraded MUC2-N, and no other protein detected in fractions 9 and 17, but not fraction 7 or 30, have any described proteolytic activity (Fig. S2, *B–D*).

Immunoblotting and MS of the gel bands from cleavage reactions revealed that the larger MUC2-N cleavage product at 125 kDa contained MUC2 D′–D3 and CysD, and the smaller product at 88 kDa contained both D1–D2 and D3–CysD (Fig. 4, *F* and *G*, and Table S1). The 32-kDa product was the GFP tag. N-terminal labeling by (*N*-succinimidyloxycarbonylmethyl)tris(2,4,6-trimethoxyphenyl)phosphonium bromide (TMPP) prior to tryptic digestion identified three possible cleavage sites: IRL^754^↓^755^IGQ, TAL^774^↓^775^ATS, and VIQ^940^↓^941^RDE (Figs. 4*D* and S3 and S4). The cleavage at IRL^754^↓^755^IGQ lies at the interface between the E2 and TIL′ domains, TAL^774^↓^775^ATS lies within the TIL′ domain, and VIQ^940^↓^941^RDE lies in the VWD3 domain.

When analyzed in nonreducing conditions, MUC2-N was mainly found in a high-molecular-mass trimer complex as described before (22) (Figs. 4*H*, ***, and S2*E*). After CLCA1 cleavage, MUC2-N was disrupted into two fragments at 72 and 250 kDa, respectively. The 250-kDa fragment was detected with a D3-recognizing MUC2 antibody and thus mostly likely corresponds to a trimer of D′–D3 and CysD, whereas the 72-kDa fragment likely contains monomers of D1–D2 (Fig. S2*E*). Based on the described disulfide bonds in the N terminus of VWF, we believe that the CLCA1-mediated cleavage at IRL^754^↓^755^IGQ causes the separation of D1–D2 from downstream MUC2 as the cleavage products from this site would not be bridged by cysteine bonds (Fig. S5*A*).

### Muc2 analysis in WT and Clca1^−/−^ animals

**Figure 5.**
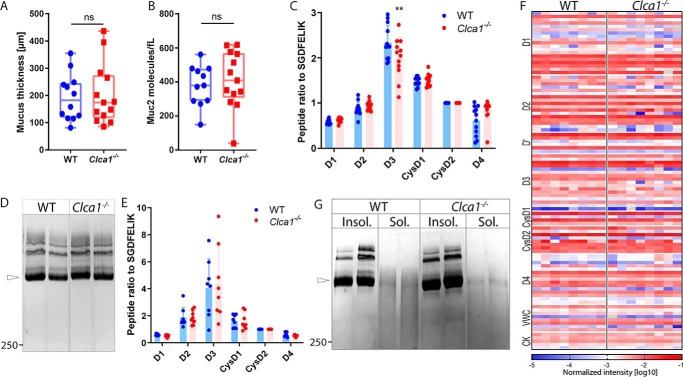
**Muc2 analysis in WT and *Clca1*^−/−^ colon.** *A*, measurement of total (inner + outer) mucus layer thickness in WT and *Clca1*^−/−^. *B*, absolute quantification of Muc2 in colonic mucus samples from WT and *Clca1*^−/−^ total mucus. *C*, Muc2 peptide ratios in relation to the CysD2 domain peptide SGDFELIK in mucus samples from WT and *Clca1*^−/−^ colon determined by MS-based absolute quantification. *D*, Muc2 in reduced mucus from WT and *Clca1*^−/−^ colon separated by SDS-UAgPAGE and stained with Alcian blue. *Open arrowhead*, monomeric Muc2. *E*, Muc2 peptide ratios in relation to the CysD2 domain peptide SGDFELIK in semipurified Muc2 from WT and *Clca1*^−/−^ colon determined by MS-based absolute quantification. *F*, heat map representation of the normalized MS-based intensities (log_10_) of Muc2 peptides in semipurified samples from WT and *Clca1*^−/−^ mice. Different regions of the Muc2 protein are indicated to the *left* of the heat map. *G*, reduced GuHCl-insoluble (*Insol.*) and -soluble (*Sol.*) mucus fractions from WT and *Clca1*^−/−^ colon separated by SDS-UAgPAGE and stained with Alcian blue. *Open arrowhead*, monomeric Muc2. *Boxes* show median with range. Bar graphs represent mean ± S.D. (*error bars*). *n* = 12–13 (*A–C*), 8 (*D–F*), or 4 (*G*). **, *p* > 0.01; *ns*, not significant. Significance was determined by unpaired *t* test (*A* and *B*) or two-way analysis of variance followed by Šídák's multiple comparison test (*D* and *E*). *Numbers* to the *right* of the blots denote the determined molecular mass (in kDa).

As all identified cleavage sites lie after the D1–D2 assemblies, we hypothesized that CLCA1-mediated cleavage leads to the removal and further degradation of D1–D2 *in vivo*. To confirm this, we performed filter-aided sample preparation (FASP) absolute quantification of Muc2 in total mucus (which comprises inner and outer mucus) from WT and *Clca1*^−/−^ colon using selected peptides from different MUC2 domains. Neither the mucus thickness nor the overall Muc2 abundance per volume was altered in Clca1-deficient mice compared with WT (Fig. 5, *A* and *B*). Furthermore, no difference between the genotypes was observed in the ratio between peptides from different Muc2 domains including D1 or D2 (Fig. 5*C*). However, as the analysis was performed on total mucus, it was possible that peptides from D1–D2 remained in the mucus after the putative cleavage by CLCA1. To further test this, Muc2 was semipurified by gel electrophoresis of extensively PBS-washed and reduced mucus samples using an SDS-UAg polyacrylamide gradient gel to remove nonattached fragments. The Muc2 migration pattern on SDS-UAgPAGE was indistinguishable between WT and *Clca1*^−/−^ mucus, and no differences in peptide ratio were observed between genotypes (Fig. 5, *D* and *E*). To rule out that this was not an effect of the peptides chosen for absolute quantification, the relative abundance of all identified Muc2 peptides was analyzed without identifying any difference between WT and *Clca1*^−/−^ (Fig. 5*F*). Several isopeptide cross-links have been identified between the Muc2 domains, including D1–D2 and downstream domains that possibly affect the solubility of Muc2 in chaotropic salts (26), but neither the Muc2 reduction sensitivity nor solubility in guanidinium chloride (GuHCl) appeared altered in *Clca1*^−/−^ mucus compared with WT (Fig. 5*G*).

**Figure 6.**
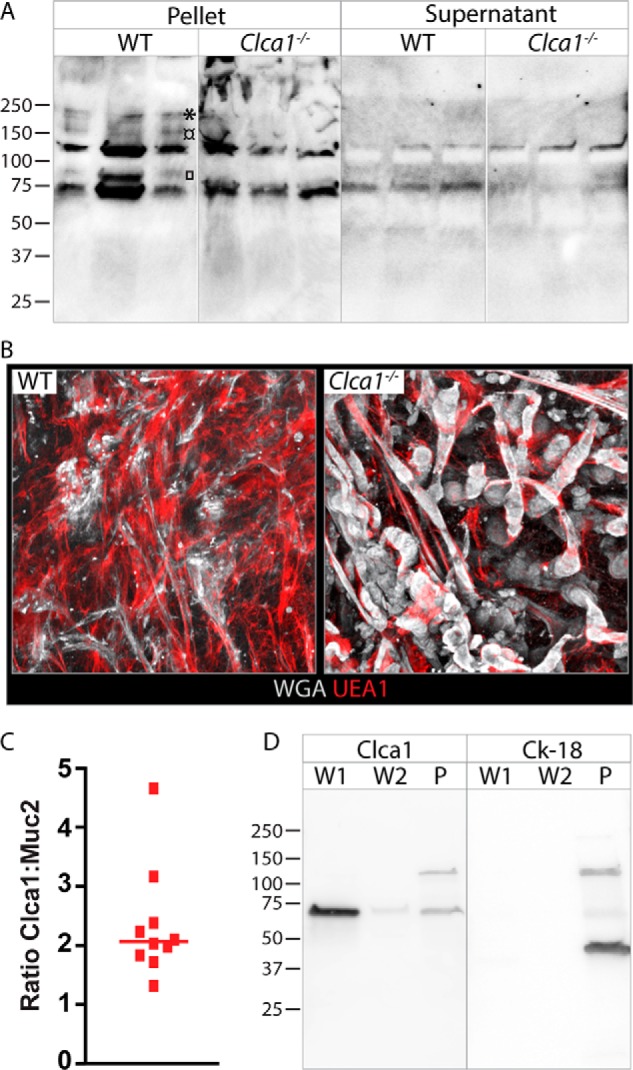
**Effect of Clca1 deficiency on colonic mucus.** *A*, immunoblot of Muc2 degradation products in PBS-washed mucus pellets or supernatant from WT or *Clca1*^−/−^ colon. □, ¤, and * denotes 85-, 160-, and 200-kDa bands, respectively. *B*, *x*/*y*-projection of the outer mucus layer in WT and *Clca1*^−/−^ colon visualized using fluorescently conjugated UEA1 (*red*) and WGA (*gray*). *C*, ratio of normalized protein intensity between Clca1 and Muc2 in mouse colonic mucus determined by MS. *D*, immunoblot of the first and second PBS washes of mucus (*W1* and *W2*, respectively) and the remaining mucus pellet (*P*) separated by SDS-PAGE after reduction, with detection of Clca1 and cytokeratin-18 (*Ck-18*). Molecular mass references are given in kDa.

To further investigate differences in D1–D2 post-translational processing by Clca1, we analyzed degradation products of Muc2 in both the mucus pellet and the soluble material isolated by centrifugation using standard gradient gels. The MUC2-N3 antibody revealed additional bands in WT compared with *Clca1*^−/−^ mucus pellet samples at ∼85, 160, and 200 kDa (Fig. 6*A*). Of these, only the 85-kDa band could be faintly detected in the soluble material. However, the ratio between the bands at 75 and 130 kDa detected in the supernatant was altered in *Clca1*^−/−^-derived samples, further indicating altered Muc2 N-terminal processing.

Alterations in Muc2 processing likely impact the mucus structure, and we thus investigated the mucus structure in WT and Clca1-deficient mice using lectin-based *ex vivo* imaging of unflushed colonic mucus. In contrast to the fine netlike structures seen at the mucus surface in WT colon, Clca1-deficient mice had a markedly altered mucus structure with large rope-like formations, indicating incomplete unfolding of the Muc2 oligomers (Fig. 6*B*). The effect of Clca1 deficiency was more noticeable in the unflushed mucus than in flushed mucus (17).

As we have previously shown, CLCA1 is one of the most abundant proteins in the colonic mucus, and investigation of the relative abundance of Clca1 to Muc2 in mouse colonic mucus revealed that it is close to 2:1, which is higher than expected for an enzyme-to-substrate ratio (Fig. 6*C*) (17). A possible explanation is that CLCA1 is bound to MUC2 in the mucus to be able to exert its proteolytic function. However, immunoblotting of mucus PBS washes and mucus pellet revealed that the vast majority of Clca1 was found in the first mucus wash, indicating that it is not bound to Muc2 (Fig. 6*D*). Some Clca1 was detected also in the mucus pellet, but the presence of the 130-kDa immature Clca1 indicated that this was due to cellular contaminations in the mucus pellet, which was confirmed by the concomitant detection of the intracellular goblet cell protein cytokeratin-18.

## Discussion

**Figure 7.**
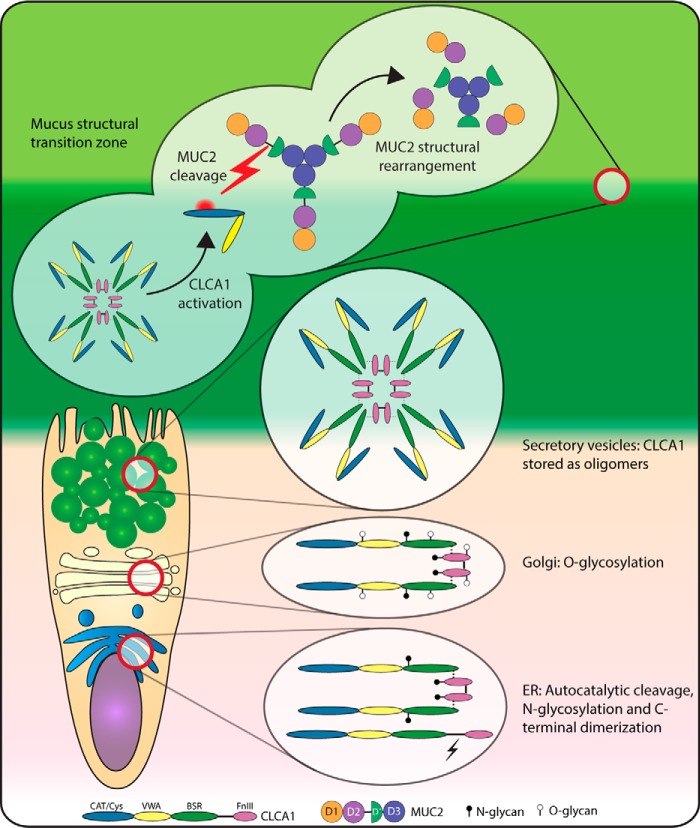
**Schematic summary.** CLCA1 undergoes full autocatalytic cleavage in the ER where afterward the C- and N-terminal products remain noncovalently associated. C-terminal CLCA1 also forms disulfide-linked dimers. *N*-Glycans are attached (*filled pins*). *O*-Glycans (*open pins*) are attached in the Golgi. CLCA1 is stored and secreted as oligomers, likely octamers, but a yet-undefined mechanism releases the CAT/Cys + VWA domains from CLCA1, which possibly activates the extracellular proteolytic activity of CLCA1. Active CLCA1 is able to cleave off D1–D2 from MUC2 and thereby mediates structural rearrangement of the mucus.

We have investigated the biochemical processing of CLCA1 in the colonic epithelium and mucus and observed that CLCA1 is glycosylated, and its C terminus forms dimers, which can further interact with the N-terminal cleavage product to form noncovalent oligomers. Besides the predicted C- and N-terminal cleavage products, we also observed a previously undescribed N-terminal cleavage product containing the CAT/Cys + VWA domains. We were further able to show that CLCA1 can process N-terminal MUC2 by proteolysis (Fig. 7).

The CLCA1 C terminus was found as disulfide-linked dimers that remained associated with the N-terminal CLCA1 cleavage product by noncovalent interactions in colonic mucus and epithelium. Furthermore, analysis of CLCA1 under native conditions indicated that CLCA1 can build large noncovalent oligomers both *in vitro* and *in vivo*. Clca1 complexes have been observed previously but were not studied in detail (28). The function of CLCA1 oligomerization remains unknown but could, as for many other oligomers, confer stability to the protein. Further characterization of the oligomeric state of CLCA1 under different conditions would shed light on external factors, such as pH or redox potential, that affect the CLCA1 structure.

Both the N- and C-terminal parts of CLCA1 were glycosylated with *N*- and *O*-linked glycans, which was also predicted from the sequence. As for many other secreted proteins, glycosylation of CLCA1 likely promotes folding and secretion and enhances solubility of the protein. However, as has been described for other proteases, glycosylation could also modulate interactions and proteolytic activities as well as affect CLCA1 oligomerization (29).

The function of CLCA1 self-cleavage is so far unknown. As previously suggested by Yurtsever *et al.* (8), the autocatalytic cleavage of CLCA1 may control the activity of CLCA1 as a calcium-activated chloride channel regulator. However, uncleaved CLCA1 is not detected *in vivo*, either in mucus or in epithelium, indicating that the cleavage occurs early and efficiently in the intracellular secretory pathway. The cleavage does not appear crucial for secretion as uncleaved material can be secreted from CLCA1-overexpressing cells (8, 10, 17). Neither does the cleavage seem crucial for proteolytic activity as shown previously where CLCA1 lacking this cleavage site was found to be proteolytically active (17).

The C-terminal cleavage product of CLCA1 has not previously been studied in great detail as it was thought to be unstable after secretion (14). However, we were able to readily detect the cleaved C terminus in both mucus and epithelial tissue. Furthermore, the C-terminal product was largely resistant to limited proteolysis, indicating that it is a structured domain, in line with the prediction of an FnIII domain. The truncated CLCA1 protein with an intact N terminus but absent C terminus is retained in the ER, indicating that the C terminus is needed for correct processing in the ER and concomitant secretion of CLCA1. Furthermore, the CLCA1 fraction with high MUC2-processing activity lacked detectable levels of C-terminal CLCA1. It is thus possible that C-terminal interactions with the N terminus directly inhibit its proteolytic activity and thus function as a regulator. However, our results indicate that a second N-terminal cleavage product harboring the CAT/Cys + VWA domains is the proteolytically active fraction of CLCA1. Therefore, it is also possible that the C-terminal part regulates CLCA1 proteolytic activity by blocking the cleavage that releases the active N-terminal fragment.

Characterization of the proposed CAT/Cys + VWA product is difficult due to its tendency to disappear after freeze/thaw cycles, indicating that it is more unstable than the full N-terminal product. As an example, the activity of CLCA1 fraction 9 used for the MUC2 *in vitro* cleavage assay was markedly decreased when the experiment was repeated, and the 55-kDa band was concomitantly lost, indicating that the CAT/Cys + VWA domains were indeed the active component in this fraction (data not shown). Decreased stability of the active product can be envisioned as a way of regulating the activity, similar to the cleaved complement factor C2a, which interestingly also harbors a VWA and a catalytic domain (30). Furthermore, we were not able to express the CAT/Cys + VWA–truncated protein as a recombinant protein in mammalian cells as it was retained intracellularly. However, the molecular mass of this fragment corresponded well to the smaller N-terminal product observed in mucus samples, further strengthening that this is indeed the CAT/Cys + VWA domains. Although this product seems unstable during freeze/thawing, it is resistant to limited proteolysis by trypsin, indicating that it forms a protected globular structure. The protease responsible for the cleavage resulting in the CAT/Cys + VWA product is still unknown.

Three CLCA1-mediated cleavage products of the MUC2 N terminus were observed at 32, 88, and 125 kDa, which we propose consist of four fragments: the GFP tag (32 kDa), a larger product of D′–D3–CysD (125 kDa), and two fragments at 88 kDa consisting of D1–D2 and D3–CysD. One of the identified cleavage sites (IRL^754^↓^755^IGQ) lies at the interface between the E2 and TIL′ domains, and proteolytic cleavage at this site would separate D1–D2 from D′–D3 as the cleavage products would not be held together by disulfide bonds, whereas the two other identified cleavage sites for CLCA1 are found between disulfide-linked parts of TIL′ and VWD3 (31). Separation of D1–D2 from the rest of the MUC2-N construct was also noted under nonreducing conditions. We thus believe that IRL^754^↓^755^IGQ is most likely the major cleavage site for CLCA1 in MUC2. The proposed cleavage between the D2 and D′ in MUC2 is similar to that previously described for Meprin β–mediated MUC2 cleavage in the small intestine as well as for furin cleavage of VWF, which indicates that this is a proteolytically susceptible part of the MUC2 and VWF structures (21, 31). Of note, sequence alignment of the different E-domains in MUC2 and VWF reveals that the E2 domain in both MUC2 and VWF lacks a disulfide bridge, which likely blocks the cleavage of CLCA1 or furin in the other E-domains (Fig. S5*B*).

No other proteins within the intestinal mucosa contain D1–D2 assemblies followed by a D′ assembly, and it is thus possible that CLCA1 is restricted to cleave MUC2. However, we cannot exclude that CLCA1 also has other substrates in intestinal mucus. FCGBP (IgGFc-binding protein), another very abundant mucus protein, does *e.g.* contain several D assemblies and could thus be envisioned as a CLCA1 substrate, but this needs further investigation.

The airway mucins MUC5AC and MUC5B both have a similar N-terminal domain arrangement as MUC2. It is thus possible that the observed induction of CLCA1 in diseased lungs in asthma, cystic fibrosis, and COPD (3) facilitates mucus structure rearrangements similar to the function of CLCA1 in the intestine. This is in line with the observation of an attached, stratified mucus layer in COPD and cystic fibrosis and mouse model of chronic lung disease similar to that in colon (32). In both colon and diseased lung, mucus will separate bacteria from the epithelial cells. Such mucus layers need to be turned over and detached, processes where the proteolytic activity of CLCA1 might have an important role.

As the function of the D1–D2 assemblies in secreted MUC2 is unknown, we can only speculate on the implication of this cleavage. In the case of Meprin β, proteolysis of MUC2 in the small intestine was suggested to release MUC2 from the epithelium to detach the mucus. In colon, the inner mucus remains attached to the epithelium, whereas the outer mucus layer is easily removable (33). As we have hypothesized that CLCA1 is involved in the transition from inner to outer mucus in colon (17), it is possible that the D1–D2 assemblies are responsible for mucus attachment and that CLCA1 is involved in the detachment of the mucus similar to Meprin β. However, Meprin β cleavage of MUC2 was shown to be pH-dependent, whereas this was not the case for CLCA1.

Intracellularly D1–D2 is involved in the packing of MUC2 by forming tight, concatenated rings together with D′–D3 that hold the MUC2 oligomers together (24). Increased pH and removal of Ca^2+^ upon secretion induce conformational changes that break the interaction between D1–D2 and D′–D3, which aids the unfolding of the MUC2 sheets. Removal of D1–D2 by CLCA1 might thus facilitate unfolding or stabilize the unfolded state, and thereby expansion of MUC2, by speeding up this process. Furthermore, in VWF, the D1–D2 assembly is intracellularly cleaved off as a propeptide by furin where after D1–D2 remains associated with the mature VWF and stabilizes intracellular assembly (27). It is thus possible that MUC2 D1–D2 likewise stabilize the structure after secretion, *e.g.* by preventing proteolytic events in the MUC2 structure by steric hindrance. CLCA1 could thus be envisioned to remove this blockage to allow other enzymes access to their substrate site, which would start to degrade/expand the mucus structure. Lastly, the D3 trimer has been suggested to be able to form hexamers after secretion, which would give a new interaction surface between MUC2 oligomers (25). However, this was only tested in fragments lacking D1–D2, and it is possible that D1–D2 prevents this interaction and needs to be removed for the interaction to take place.

To seek *in vivo* confirmation of the observed *in vitro* CLCA1-mediated MUC2 cleavage, mucus from WT and *Clca1*^−/−^ animals was analyzed. No difference could be detected either in total mucus thickness or Muc2 concentration in the mucus from *Clca1*^−/−^ animals compared with WT, indicating that MUC2 expansion is not altered as this should lead to changes in these parameters. However, the absolute quantification generated large variability; thus, small changes would not be detected using this method. Additionally, as we have previously shown, compensatory mucus-processing mechanisms in *Clca1*^−/−^ animals can possibly mask the phenotype (17). We also could not detect any difference in D1–D2 peptide abundance between genotypes, indicating that D1–D2 is not further degraded after cleavage and remains in the mucus. D1–D2 peptides were abundantly identified also after reducing semipurification of MUC2, either due to cross-linking of D1–D2 to downstream domains or because the Muc2 analyzed with this method is intact, uncleaved Muc2. However, by looking at smaller cleaved fragments of MUC2 in the mucus, a different band pattern was seen in *Clca1*^−/−^ mice compared with WT, strengthening the *in vitro* results. The alteration in MUC2 D1–D2 processing in *Clca1*^−/−^ animals seems to have a large impact on the mucus layer structure, which was much more compact in *Clca1*^−/−^ colon, indicating that CLCA1-mediated cleavage of the MUC2 N terminus is indeed involved in the expansion or unfolding of MUC2 and that this is crucial for forming the netlike sheets seen in WT mucus.

CLCA1 is very highly abundant in intestinal mucus from both human and mouse (17). More detailed investigation of the ratio of Clca1 to Muc2 indicated a higher enzyme-to-substrate ratio than normally expected, with a 2:1 ratio of Clca1 to Muc2. The activity of CLCA1 is thus probably very tightly regulated. Although Clca1 was not found to be associated with Muc2 in mucus, it is possible that a high level of enzyme is required to reach its target in the relatively diluted mucus or that CLCA1 has structural properties required for mucus organization. Thus, the possibility that CLCA1 serves additional, yet unexplored functions in the mucus that require high levels of the protein cannot be excluded.

The identification of MUC2 as a substrate for the CLCA1 metalloprotease marks a breakthrough in the understanding of CLCA1 function. However, several questions remain unanswered such as how CLCA1 activity is regulated and how the proteolytic processing of the D1–D2 domain in MUC2 alters the mucus structure. By characterizing what we suggest is the active CLCA1 product, we hope to bring this research forward with future studies to better understand the function of CLCA1 in intestinal mucus.

## Experimental procedures

### Animals

*Clca1*^−/−^ (Clca1^tm1Htzm^, RRID:MGI: 3802573) (34) and WT mice on C57Bl/6N background from homozygous breeding colonies were used in full compliance with Swedish animal welfare legislation and approved by the Swedish Laboratory Animal Ethical Committee in Gothenburg, Sweden (numbers 280-12 and 73-15). *Clca1*^−/−^ and WT mice were kept in a specific pathogen-free facility in single ventilated cages with food and water *ad libitum* and 12-h light/dark cycles. Isoflurane was used for anesthesia followed by cervical dislocation for sacrificing the mice. Animals of both genders were used at an age of 8–16 weeks.

### Human samples

Sigmoid colon biopsies for mucus and epithelial collection were obtained from patients referred for colonoscopy at Sahlgrenska University Hospital, Gothenburg, Sweden, in compliance with the human research ethical committee in Gothenburg, Sweden (040-08), and the Declaration of Helsinki. Patients with normal intestinal macroscopy were included in the study after informed consent. Biopsies were obtained as described previously (33).

### Mucus and epithelium sample collection and preparation

Mouse colonic mucus and epithelial cell samples for SDS-PAGE were scraped in ice-cold PBS containing 2× cOmplete EDTA-free protease inhibitor mixture (Roche Applied Science) and collected in ice-cold lysis buffer (50 mm Tris-HCl, pH 8.0, 150 mm NaCl, 0.1% (v/v) Triton X-100) containing 2× cOmplete EDTA-free protease inhibitor mixture. From this point, all samples were kept on ice or at 4 °C. All samples were rotated for 1 h prior to homogenization of remaining clumps with a plastic micropestle (Sigma-Aldrich). The samples were centrifuged at 1000 × *g* for 5 min. Samples were either reduced in sample buffer (50 mm Tris-HCl, pH 6.8, 2% SDS, 10%glycerol) containing 100 mm dithiothreitol (DTT) or denatured in sample buffer only at 95 °C for 5 min before loading and running SDS-PAGE as described below.

Mouse samples for native PAGE were similarly collected in 1× NativePAGE^TM^ sample buffer (Novex®), homogenized with a plastic micropestle, and centrifuged at 1000 × *g* for 5 min. G-250 was added to the samples according to the manufacturer's recommendation before loading on gels. Isolation of GuHCl-insoluble and -soluble mucus fractions was performed as described previously (35).

Human sigmoid colon biopsies were pinned down on a silica gel plate in a small volume of ice-cold Krebs transport buffer (33) with the mucosal side facing up. Mucus was scraped off and collected with 2× cOmplete EDTA-free protease inhibitor mixture. Remaining epithelium was homogenized in 150 μl of Krebs transport buffer supplemented with 2× cOmplete EDTA-free protease inhibitor mixture using a plastic micropestle. The homogenate was spun at 14,000 × *g* for 10 min at 4 °C, and the pellet was discarded. Samples for SDS-PAGE were prepared as described for mouse samples above. Human mucus and epithelium samples as well as medium containing rCLCA1 were desalted using Amicon Ultra 0.5-ml 30 kDa cutoff centrifugal filters (Merck) before addition of G-250 and NativePAGE sample buffer.

### Expression and purification of recombinant CLCA1

pcDNA3.1 vectors containing the human CLCA1 sequence (11) with or without a C-terminal His tag and with or without alterations (see below) were used for mammalian expression of rCLCA1. Vectors were transiently transfected into CHO-K1 cells (for small-scale production) or FreeStyle CHO-S cells (for large-scale production), and spent medium was collected at 72 h post-transfection. Cells and debris were removed by centrifugation. The remaining supernatant was either directly mixed with reducing or nonreducing sample buffer and analyzed by SDS-PAGE and immunoblotting or dialyzed against buffer A (20 mm Tris-HCl, pH 8) for downstream ion-exchange chromatography or against PBS for His tag–aided purification. Ion-exchange chromatography was performed on a Mono Q^TM^ 5/50 GL anion-exchange column (5 mm × 50 mm; GE Healthcare) at 0.2 ml/min using an ÄKTA HPLC (GE Healthcare). Bound components were eluted in a linear gradient of 0–100% buffer B (20 mm Tris-HCl, pH 8, 1 m NaCl). A HiTrap chelating column with cobalt was used for His purification, and eluted fractions with rCLCA1 were pooled and dialyzed against PBS. All eluted fractions were analyzed by reducing SDS-PAGE and Western blotting. Protein concentrations were determined with a BCA Protein Assay kit (Pierce).

### Mutagenesis

Truncated versions of CLCA1 were generated by inserting a His_6_ tag and a stop codon at the desired locations using a QuikChange site-directed mutagenesis kit (Agilent Technologies) according to the manufacturer's instruction with 50 ng of parental vector DNA. CLCA1 1–300 (300) was introduced using forward primer 5′-TCC CAC CTT CTCA TTG CTG CAT CAT CAC CAT CAC CAC TAG TCA GAT TGG ACA AAG AAT TG-3′, CLCA1 1–477 (477) was introduced using 5′-CTT TTG GGG CCC TTT CAT CAC ATC ATC ACC ATC ACC ACT AGT GGA AAT GGA GCT GTC-3′, and CLCA1 1–695 was introduced using 5′-TAC CCC AGC AGA GTG GAC ATC ATC ACC ATC ACC ACT AGT GCA CTG TAC ATA CCT-3′. The reverse complementary sequences to the forward primers were used as reverse primer in all cases. Purified plasmids were sequenced to confirm the mutations.

### Medium and cell lysate preparation from small-scale transfections

cOmplete EDTA-free protease inhibitor mixture was added to pooled and collected medium from transfected cells. Transfected cells were washed with PBS before treatment with ice-cold lysis buffer containing 1× cOmplete EDTA-free protease inhibitor mixture for 30 min on ice. The resulting lysate was briefly ultrasonicated and then centrifuged for 10 min at 14,000 rpm at 4 °C.

### Glycosylation analysis

Purified rCLCA1 was treated for 3 h at 37 °C with PNGaseF (Roche Applied Science) and a combination of sialidases (SialEXO cleaves α2–3, α2–6, and α2–8; Genovis, Lund, Sweden) and *O*-glycosidase (New England Biolabs) after denaturation in 0.1% SDS at 95 °C for 5 min and subsequent addition of Triton X-100 to 1% final concentration.

For EndoH analysis, lysates from transfected cells were reduced in sample buffer containing DTT (100 mm) at 95 °C for 5 min. The pH was adjusted to 5.5 by adding sodium citrate. After addition of EndoH (Roche) or PBS as control, the samples were incubated at 37 °C overnight before analysis by SDS-PAGE and immunoblotting.

### Gel electrophoresis

10% SDS-PAGE with 4% stacking gel or Mini-PROTEAN TGX 4–20% gels (Bio-Rad) were used for gel separations unless otherwise stated. Samples were boiled at 95 °C in SDS sample buffer with or without 100 mm DTT as indicated (reduced and nonreduced, respectively) before separation. The NativePAGE Novex Bis-Tris gel (4–16%) system was run according to the manufacturer's recommendations. Muc2 oligomers were analyzed by separating DTT-reduced mucus samples by SDS-UAgPAGE prepared as described before (36). SYPRO^TM^ Ruby Protein Gel Stain (Invitrogen) was used for detection of proteins in SDS-polyacrylamide gels according to the manufacturer's quick stain protocol. The gel was subsequently incubated in SDS-PAGE running buffer for 20 min at room temperature and blotted to PVDF as outlined below. Alcian blue was used for detection on proteins separated by SDS-UAgPAGE.

### Western blotting

SDS-PAGE gels were blotted to PVDF membranes by semidry Western blotting using 1× PVDF blotting buffer (48 mm Tris, 39 mm glycine, 1.3 mm SDS) plus 20% MeOH at 2 mA/cm^2^ unless otherwise stated. 5% milk powder in PBS-Tween 20 (0.1%) was used as blocking solution. The membranes were washed repeatedly in PBS-Tween 20 (0.1%) between all steps after addition of primary antibody. Primary antibodies were used as indicated according to Table 1. Alkaline phosphate– or horseradish peroxidase–conjugated secondary antibodies were used for either colorimetric detection with nitro blue tetrazolium and 5-bromo-4-chloro-3′-indolyl phosphate or electrochemiluminescence detection in a LAS-4000 Mini (Fujifilm). Molecular mass analysis of detected bands in Western blots was performed in ImageJ by comparing the retardation factor of the detected bands with those of the molecular weight standard (Precision Plus Protein^TM^ Dual Color/Unstained/WesternC Standard, Bio-Rad).

**Table 1:** **Antibodies used for detection of CLCA1 or MUC2**

Antibody	Species reactivity	Epitope	Domain	Dilution	Ref./source
**CLCA1**					
Clca3 3-ar	Mouse, human	866–879	C-terminal/FnIII	1:1,000	10
ab46512	Mouse	253–267	VWA	1:1,000	Abcam
ab129283	Human	677–776	C-terminal/FnIII	1:1,000	Abcam
Trx-hCLCA1	Human	22–124	N-terminal/CAT	1:2,000	11
ab180851	Human	No data	VWA	1:10,000	Abcam
**MUC2**					
MUC2-N3	Human	1167–1180	D3	1:1,000	42

### His pulldown

Spent medium from a small-scale transfection of C-terminally His-tagged CLCA1 was used for Dynabeads^TM^ His-tag isolation and pulldown (Thermo Fisher Scientific) according to the manufacturer's His-tag isolation instructions. The eluate was reduced with DTT and analyzed by gel electrophoresis and immunoblotting.

### Limited proteolysis

Purified rCLCA1 was incubated with trypsin at a 100:1 ratio in PBS at 37 °C. Samples for SDS-PAGE were taken at *t* = 0, 10, 30, 60, and 120 min; mixed with SDS-PAGE sample buffer containing DTT; boiled at 95 °C for 5 min; and subsequently separated by SDS-PAGE and immunoblotted.

### Gel filtration

25 μg of affinity-purified rCLCA1 with C-terminal His was injected into a 2.2-ml Superose 6 Increase 3.2/300 size-exclusion chromatography column (GE Healthcare) with a size-exclusion range of 5000–3,000,000 Da using an Ettan^TM^ LC system (GE Healthcare) and the software Unicorn 5.01 (GE Healthcare). The system was run at a speed of 40 μl/min, eluting 1.5 column volumes of 50 mm HEPES, pH 7.4, 150 mm NaCl, collected in 100-μl fractions using a Frac950 (GE Healthcare) fraction collector and an ÄKTA BOX 900 (GE Healthcare). 5 mg/ml thyroglobulin (669,000 Da), ferritin (440,000 Da), aldolase (160,000 Da) and ovalbumin (42,700 Da) were used for column calibration and as molecular weight standards (GE Healthcare). The fractions containing rCLCA1 were determined through SDS-PAGE and Western blotting of all fractions showing a peak at 280-nm absorbance.

### MUC2 proteolysis assay

MUC2-N or MUC2-C (1–1.5 μg) (22, 23) were subjected to a proteolytic assay using two fractions from ion-exchange purification of rCLCA1 in 20 mm Tris-HCl, pH 7.5, with 150 mm NaCl and 10 mm CaCl_2_ as assay buffer with and without 5 mm EDTA. rCLCA1 from fraction 17 (1 μg) or an equal volume of fraction 9 was used. The reactions were incubated for 18 h at 37 °C, mixed with SDS-PAGE sample buffer containing DTT, boiled at 95 °C for 5 min, and subsequently separated by SDS-PAGE. Gels were imaged by SYPRO Ruby protein gel stain or blotted to PVDF membrane for subsequent immunoblotting. For reactions at pH 6, MUC2-N was preincubated in 20 mm MES, pH 6, with 150 mm NaCl for 2 h on ice before addition of rCLCA1.

### In-gel digestion and TMPP labeling

Bands of interest were excised from SDS-PAGE gels and processed as described before (37) or according to the TMPP labeling protocol (38).

### Absolute Muc2 quantification and Muc2 peptide analysis

Mucus for absolute Muc2 quantification and proteome analysis was sampled from WT and Clca1-deficient mice. The colonic tissue was left unflushed (to study the total mucus, including the outer mucus layer, fecal material was gently removed after opening of the tissue), opened, and pinned flat in ice-cold Krebs transport buffer on a silica gel plate for ∼2 min to relax the tissue. The specimens were then mounted in a horizontal mucus measurement chamber (detailed in Ref. 33) with static basolateral Krebs transport buffer. Black 10-μm polystyrene Polybead microspheres (Polysciences, Germany) were added apically and left to sediment for 2 min before the apical chamber was filled. The mucus thickness was measured (33), and the mucus was collected immediately by suction and very gentle scraping of the epithelium and the rim of the chamber using an in-house microspatula (1.5 mm). 2× cOmplete EDTA-free protease inhibitor mixture was added to the samples.

Subsequent FASP of the samples has been outlined in detail previously (39). In brief, samples were prepared according to a FASP protocol adopted from Wiśniewski *et al.* (40). Heavy peptides (SpikeTides TQL, JPT Peptide Technologies, Berlin, Germany) for Muc2 absolute quantification (D1, TFDGDVYR; D2, TVVLLTDDK; D′, DLYSSGESIK; D3, IFIGGTELK; CysD1, LSWEELGQK; CysD2, SGDFELIK; D4, HETQEVQIK; 100 fmol each) were added before trypsin digestion.

For absolute quantification of semipurified Muc2, mucus from distal colon was scraped in 150 μl of ice-cold PBS containing 1× cOmplete EDTA-free protease inhibitor mixture and 10 mm EDTA. The sample was washed in 1 ml of ice-cold PBS five times with intermittent centrifugation at 17,000 × *g* for 20 min. The final pellet was reduced in sample buffer containing 800 mm DTT for 15 min at 95 °C before SDS-UAgPAGE. Muc2 was stained with Alcian blue, and in-gel trypsin digestion with ProteaseMAX^TM^ surfactant (Promega) was performed in excised Muc2 bands according to the manufacturer's instructions. Heavy Muc2 peptides were added before trypsin digestion.

### Mass spectrometry and data analysis

Peptides were cleaned with StageTip C_18_ columns prior to analyzing with EASY-nLC (Thermo Scientific) connected to a Q-Exactive (Thermo Scientific) mass spectrometer. Mass spectrometry was run in data-dependent mode for most of the analysis; targeted MS was used for the absolute quantification of Muc2. Peak lists were identified with MaxQuant software version 1.5.7.4 (41), PEAKS software version 8.5, or Mascot version 2.3.02 by searching against an in-house human and mouse mucin database version 2.0 (43) and mouse UniProt database. Searches were performed with semispecific tryptic specificity, a maximum of two missed cleavages, carbamidomethylation (57.02) as fixed modification, and oxidation of methionine (15.99) as variable modification. TMPP label (572.18) on the N terminus, lysine, and tyrosine was included as a variable modification in the case of N-terminal TMPP labeling.

Skyline software version 3.6 was used for absolute quantification analysis. Relative quantification was based on normalized intensities.

### Ex vivo lectin staining

*Ex vivo* lectin staining of mucus from unflushed WT and *Clca1*^−/−^ colon using rhodamine-conjugated UEA1 and Alexa Fluor 647–conjugated WGA (Vector laboratories) at 50 μg/ml was performed as described before (17).

### Sequence alignment and glycosylation prediction

NetOGlyc 4.0 and NetNGlyc 1.0 were used for glycosylation site prediction of human CLCA1 (UniProt accession number A8K7I4). Sequences for human VWF (UniProt accession number L8E853), human MUC2 (NCBI accession number AZL49145.1), and mouse Muc2 (43) (UniProt accession number Q80Z19) were aligned using Clustal Omega multiple sequence alignment tool.

## Author contributions

E. E. L. N., G. C. H., and M. E. V. J. conceptualization; E. E. L. N., L. A., E. E., G. C. H., and M. E. V. J. data curation; E. E. L. N. and L. A. software; E. E. L. N., L. A., E. E., G. C. H., and M. E. V. J. formal analysis; E. E. L. N., L. A., and M. E. V. J. validation; E. E. L. N., L. A., and E. E. investigation; E. E. L. N. and M. E. V. J. visualization; E. E. L. N., L. A., and E. E. methodology; E. E. L. N. writing-original draft; E. E. L. N. and M. E. V. J. project administration; E. E. L. N., L. A., E. E., G. C. H., and M. E. V. J. writing-review and editing; G. C. H. and M. E. V. J. resources; G. C. H. and M. E. V. J. supervision; G. C. H. and M. E. V. J. funding acquisition.

## Supplementary Material

Supporting Information

## Footnotes

2 The abbreviations used are:

CLCA: calcium-activated chloride channel regulator
hCLCA: human CLCA
mClca: murine Clca
MUC2: mucin 2
VWA: von Willebrand domain type A
VWD: von Willebrand D
CysD: cysteine domain
PTS: proline, threonine, and serine–rich
CK: cysteine-knot
TIL: trypsin inhibitor–like
E: fibronectin type 1–like
Cys: cysteine-rich region
CAT: catalytic domain
MIDAS: metal ion–dependent adhesion site
BSR: β-sheet–rich
FnIII: fibronectin type III
E: fibronectin type I–like
ADAM: a disintegrin and metalloproteinase
VWF: von Willebrand factor
COPD: chronic obstructive pulmonary disease
PNGaseF: peptide:*N*-glycosidase F
PNA: peanut agglutinin
rCLCA1: recombinant CLCA1
aa: amino acids
EndoH: endoglycosidase H
ER: endoplasmic reticulum
TMPP: (*N*-succinimidyloxycarbonylmethyl)tris(2,4,6-trimethoxyphenyl)phosphonium bromide
FASP: filter-aided sample preparation
UAg: urea agarose
GuHCl: guanidinium chloride
CHO: Chinese hamster ovary
Bis-Tris: 2-[bis(2-hydroxyethyl)amino]-2-(hydroxymethyl)propane-1,3-diol
PVDF: polyvinylidene difluoride
WGA: wheat germ agglutinin.
