# Supplementary material for: Calcium-activated chloride channel regulator 1 (CLCA1) forms non-covalent oligomers in colonic mucus and has mucin 2–processing properties
Source: J Biol Chem. 2019 Sep 29;294(45):17075–89. doi: 10.1074/jbc.RA119.009940 (PMC6851300; doi:10.1074/jbc.RA119.009940)
Supplement: Supporting Information [file supp_294_45_17075__index.html]

Calcium-activated chloride channel regulator 1 (CLCA1) forms non-covalent oligomers in colonic mucus and has Mucin 2-processing properties — CLCA1 in colonic mucus — Calcium-activated chloride channel regulator 1 (CLCA1) forms non-covalent oligomers in colonic mucus and has mucin 2–processing properties — CLCA1 in colonic mucus — Supporting Information 

# Calcium-activated chloride channel regulator 1 (CLCA1) forms non-covalent oligomers in colonic mucus and has mucin 2–processing properties

## Supporting Information

- Supporting Information CLCA1 in colonic mucus - Supporting figures and tables.
