# Supplementary material for: Calcium-activated chloride channel regulator 1 (CLCA1) forms non-covalent oligomers in colonic mucus and has mucin 2–processing properties
Source: J Biol Chem. 2019 Sep 29;294(45):17075–89. doi: 10.1074/jbc.RA119.009940 (PMC6851300; doi:10.1074/jbc.RA119.009940)
Supplement: Supporting Information [file supp_RA119.009940_153731_3_supp_399426_pyh788.pdf]

Calcium-activated chloride channel regulator 1 (CLCA1) forms non-covalent oligomers in colonic mucus and has Mucin 2–processing properties

Elisabeth E.L. Nyström, Liisa Arike, Erik Ehrencrona, Gunnar C. Hansson, and Malin E.V. Johansson*

Supplemental information

Fig. S1. A: Detection of C-terminal rCLCA1 before (-) or after PNGaseF ("N") and SialEXO plus *O*-glycosidase ("O") treatement. **B:** Detection of T-antigen on untreated rCLCA1 using PNA lectin. **C:** Detection of N-terminal rCLCA1 before (-) or after PNGaseF ("N") and SialEXO plus *O*-glycosidase ("O") treatement. ¤ denotes uncleaved CLCA1. ^ and * denotes cleaved N- and C-terminal CLCA1 respectively. Molecular mass references are given in kDa.

Fig. S2
A: *In vitro* cleavage of MUC2-C by rCLCA1 separated on SDS-PAGE and stained with SYPRO Ruby. Different fractions from the rCLCA1 purification was tested (#17, and #9). Red arrows mark bands from CLCA1 fraction 17. **B-C:** *In vitro* cleavage of MUC2-N by rCLCA1 fraction #7 and #30 separated on SDS-PAGE and stained with SYPRO Ruby (B) or immunoblotted with αMUC2-N3 (C). **D:** Table with proteins detected by mass spectrometry in rCLCA1 purification fraction #9 and #17 but not in #7 and #30. **E:** Immunoblotting of i*n vitro* cleavage of MUC2-N by rCLCA1 separated on SDS-PAGE under non-reducing conditions (-DTT), using αMUC2-N3.

Fig. S3
A: Annotated spectrum with alignment, ion table and error map for N-terminally TMPP labelled peptide. IGQSCTAPK. **B:** Annotated spectrum with alignment, ion table and error map for N-terminally TMPP labelled peptide ATSKPR.

Fig S4
Annotated spectrum with alignment, ion table and error map for N-terminally TMPP labelled peptide RDEGHHVAYTTR

Fig. S5
A: Sequence alignment of E2, TIL´ and VWD3 from VWF, human MUC2 (hMUC2) and mouse Muc2 (mMuc2) with marked disulfide bridges based on disulfide bridges described for VWF [1]. CLCA1 cleavage sites are marked by red arrows. Furin cleavage site in VWF is marked by black arrow. **B:** Sequence alignment of E1, E2 and E´ from human MUC2 (hMUC2) and VWF with marked disulfide bridges based on disulfide bridges described for VWF. CLCA1 cleavage site is marked by red arrow.

Table S1.
Intensity of MUC2 peptides shown in Figure 4H with marked start and end position.

Table S2.
Intensity of MUC2 peptides shown in Figure 5F with marked start position.

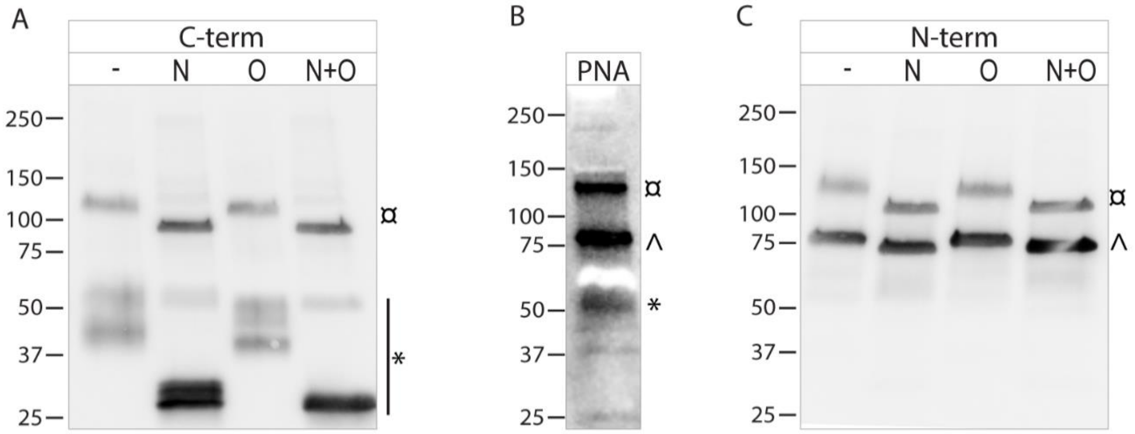

Fig. S1. A: Detection of C-terminal rCLCA1 before (-) or after PNGaseF ("N") and SialEXO plus *O*-glycosidase ("O") treatement. **B:** Detection of T-antigen on untreated rCLCA1 using PNA lectin. **C:** Detection of N-terminal rCLCA1 before (-) or after PNGaseF ("N") and SialEXO plus *O*-glycosidase ("O") treatement. ¤ denotes uncleaved CLCA1. ^ and * denotes cleaved N- and C-terminal CLCA1 respectively. Molecular mass references are given in kDa.

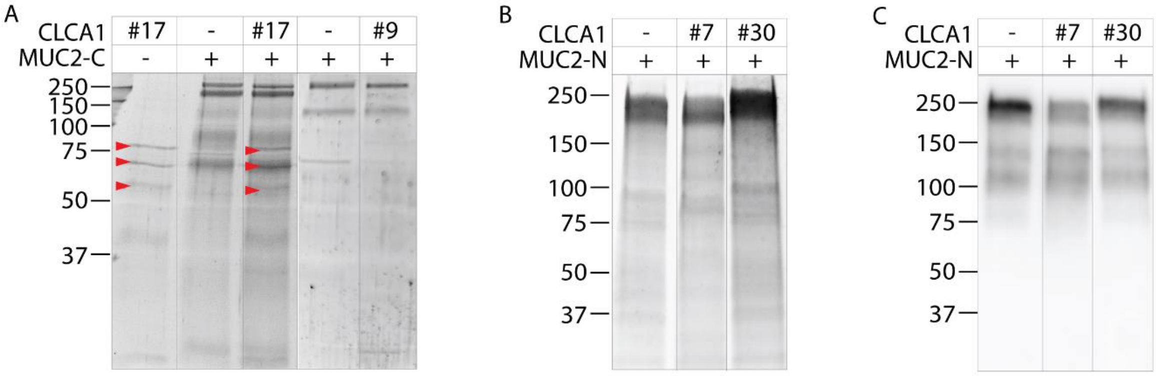

D

Gene names	Intensity #7	Intensity #9	Intensity #17	Intensity #30
CLCA1	0	1.1E+07	3.8E+08	0
ZNF445	0	1.3E+08	3.3E+08	0
FLNA	0	7.4E+06	3.9E+07	0
HSPA8;HSPA2	0	8.2E+08	2.5E+07	0
UBB;UBC;UBA52;RPS27A	0	2.1E+07	2.1E+07	0

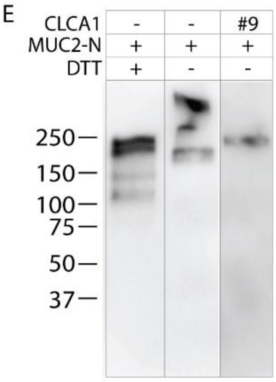

Fig. S2. A: *In vitro* cleavage of MUC2-C by rCLCA1 separated on SDS-PAGE and stained with SYPRO Ruby. Different fractions from the rCLCA1 purification was tested (#17, and #9). Red arrows mark bands from CLCA1 fraction 17. **B-C:** *In vitro* cleavage of MUC2-N by rCLCA1 fraction #7 and #30 separated on SDS-PAGE and stained with SYPRO Ruby (B) or immunoblotted with αMUC2-N3 (C). **D:** Table with proteins detected by mass spectrometry in rCLCA1 purification fraction #9 and #17 but not in #7 and #30. **E:** Immunoblotting of i*n vitro* cleavage of MUC2-N by rCLCA1 separated on SDS-PAGE under non-reducing conditions (-DTT), using αMUC2-N3.

A

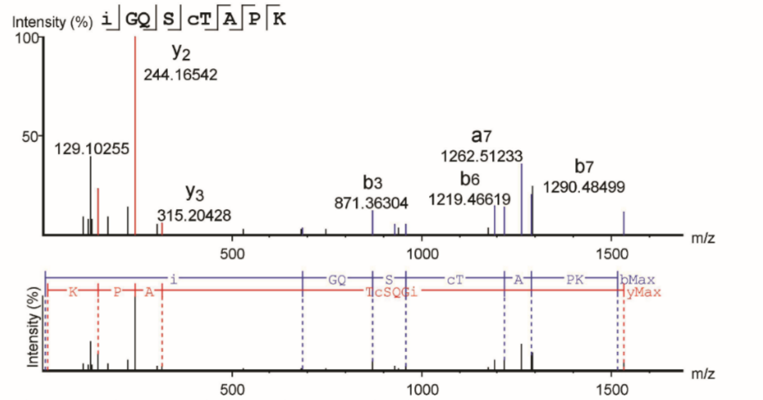

#	b	a	Seq	y	#
1	686.26233	658.27795	I(+572.18)		9
2	743.29443	715.29944	G	848.39307	8
3	871.36304	843.35797	Q	791.37158	7
4	958.37939	930.38831	S	663.31299	6
5	1118.41565	1090.42065	C(+57.02)	576.28101	5
6	1219.46619	1191.44214	T	416.25034	4
7	1290.48499	1262.51233	A	315.20428	3
8	1387.55322	1359.55823	P	244.16542	2
9			K	147.11290	1

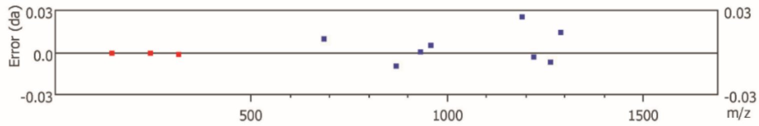

B

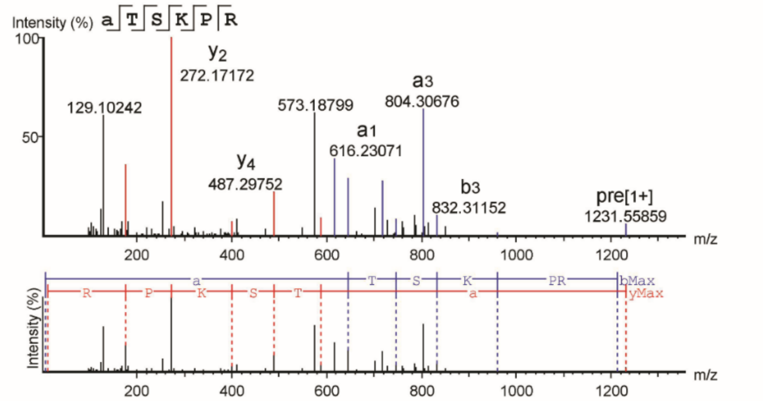

#	b	a	Seq	y	#
1	644.22510	616.23071	A(+572.18)		6
2	745.27472	717.27911	T	588.34778	5
3	832.31152	804.30676	S	487.29752	4
4	960.40344	932.40570	K	400.26556	3
5	1057.45349	1029.45850	P	272.17172	2
6			R	175.11909	1

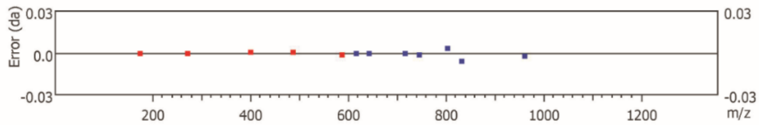

Fig. S3. A: Annotated spectrum with alignment, ion table and error map for N-terminally TMPP labelled peptide. IGQSCTAPK. **B:** Annotated spectrum with alignment, ion table and error map for N-terminally TMPP labelled peptide ATSKPR.

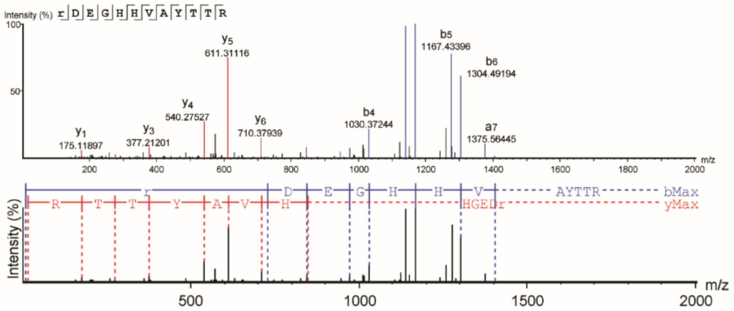

#	b	a	Seq	y	#
1	729.27972	701.29504	R(+572.18)		12
2	844.31665	816.32196	D	1285.59192	11
3	973.34808	945.35919	E	1170.56506	10
4	1030.37244	1002.38098	G	1041.52246	9
5	1167.43396	1139.43811	H	984.50098	8
6	1304.49194	1276.49512	H	847.44080	7
7	1403.56604	1375.56445	V	710.37939	6
8	1474.60437	1446.60938	A	611.31116	5
9	1637.66772	1609.67273	Y	540.27527	4
10	1738.71533	1710.72034	T	377.21201	3
11	1839.76306	1811.76807	T	276.16492	2
12			R	175.11897	1

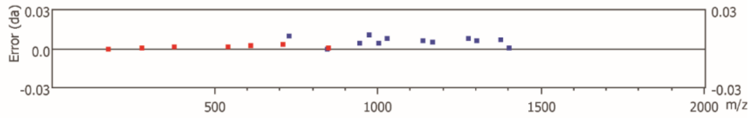

Fig S4. Annotated spectrum with alignment, ion table and error map for N-terminally TMPP labelled peptide RDEGHHVAYTTR

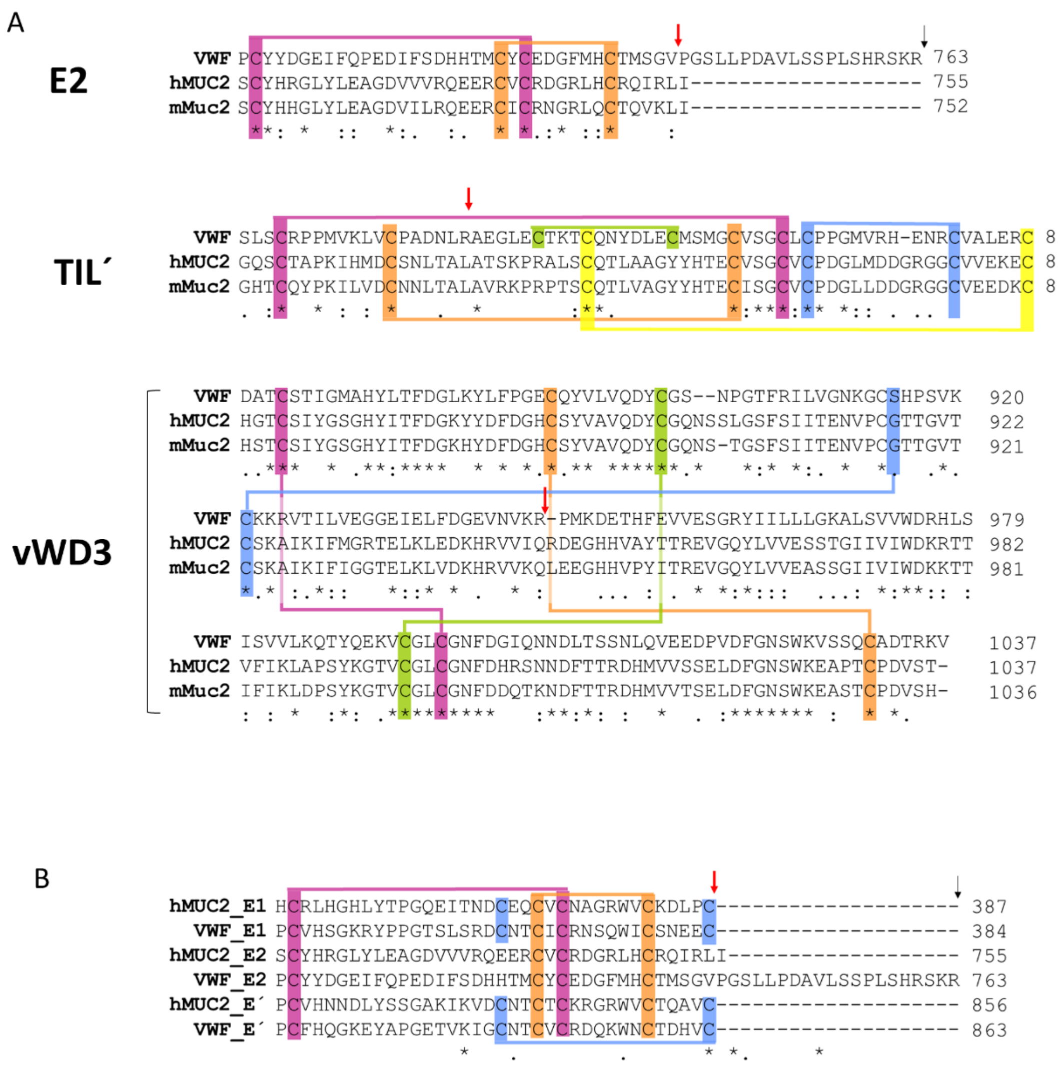

Fig. S5. A: Sequence alignment of E2, TIL´ and VWD3 from VWF, human MUC2 (hMUC2) and mouse Muc2 (mMuc2) with marked disulfide bridges based on disulfide bridges described for VWF [1]. CLCA1 cleavage sites are marked by red arrows. Furin cleavage site in VWF is marked by black arrow. **B:** Sequence alignment of E1, E2 and E´ from human MUC2 (hMUC2) and VWF with marked disulfide bridges based on disulfide bridges described for VWF. CLCA1 cleavage site is marked by red arrow.

Table S1: Intensity of MUC2 peptides shown in Figure 4H with marked start and end position.

			Log10 intensity			
**Peptide sequence**	**Start position**	**End position**	**250**	**125**	**88**	**32**
DAAQPAR	21	27	8,41	7,39	6,94	NaN
RAVR	28	31	8,34	NaN	NaN	NaN
VRSSSELQTEGR	30	41	5,82	NaN	NaN	NaN
RSSSELQTEGR	31	41	7,50	NaN	NaN	NaN
SSSELQTEGR	32	41	9,16	7,92	8,81	7,06
SSELQTEGR	33	41	7,35	7,96	9,02	7,59
SELQTEGR	34	41	8,19	7,62	8,49	7,67
ELQTEGR	35	41	NaN	NaN	6,92	NaN
QTEGR	37	41	NaN	NaN	7,40	NaN
TEGR	38	41	NaN	NaN	9,30	NaN
NVCSTWGNFHYK	48	59	7,28	6,86	7,34	NaN
TFDGDVFR	60	67	8,41	7,63	8,34	6,96
FPGLCDYNFASDCR	68	81	7,68	6,80	7,77	NaN
GLCDYNFASDCR	70	81	5,98	NaN	6,30	NaN
GSYKEFAVHLK	82	92	7,83	NaN	8,04	NaN
GSYKEFAVHLKR	82	93	7,51	NaN	7,44	NaN
EFAVHLKR	86	93	7,74	6,68	7,34	NaN
RGPGQAEAPAGVESILLTIK	93	112	NaN	NaN	6,94	NaN
GPGQAEAPAGVESILLTIK	94	112	7,60	NaN	7,34	NaN
TIKDDTIYLTR	110	120	5,66	NaN	NaN	NaN
DDTIYLTR	113	120	7,39	NaN	7,54	NaN
HLAVLNGAVVSTPHYSPGLLIEK	121	143	NaN	NaN	6,96	NaN
SDAYTKVYSR	144	153	8,26	7,40	8,02	NaN
AGLTLMWNR	154	162	7,97	7,38	7,91	NaN
EDALMLELDTK	163	173	8,04	6,94	7,84	NaN
EDALMLELDTKFR	163	175	7,10	NaN	7,17	NaN
INQPDVVCEDPEEEVAPASCSEHR	212	235	7,45	NaN	7,86	NaN
PEEEVAPASCSEHR	222	235	6,90	NaN	6,54	NaN
LLTAEAFADCQDLVPLEPYLR	241	261	8,84	7,67	8,63	7,75
AFADCQDLVPLEPYLR	246	261	7,08	NaN	NaN	NaN
QDLVPLEPYLR	251	261	6,60	NaN	NaN	NaN
CPGGDTCVCSTVAEFSR	270	286	7,67	7,19	7,92	NaN
GGDTCVCSTVAEFSR	272	286	7,14	6,61	7,29	NaN
STVAEFSR	279	286	6,68	NaN	NaN	NaN
QCSHAGGRPGNWR	287	299	7,30	NaN	7,51	6,25
PGNWR	295	299	7,38	NaN	NaN	NaN
TATLCPK	300	306	7,94	7,38	8,16	6,94
LHGHLYTPGQEITNDCEQCVCNAGR	368	392	7,62	7,19	7,80	NaN
DLPCPGTCALEGGSHITTFDGK	397	418	7,53	NaN	NaN	NaN
TYTFHGDCYYVLAK	419	432	7,71	NaN	7,53	NaN
TVVLLADK	459	466	7,92	NaN	7,85	NaN
TVVLLADKK	459	467	7,42	6,79	7,35	NaN
TVVLLADKKK	459	468	7,80	NaN	7,14	NaN
GLEGDDFK	546	553	NaN	NaN	6,36	NaN
TASGLVEATGAGFANTWK	554	571	8,15	7,51	8,02	NaN
KTETPFGR	606	613	8,75	7,96	8,57	7,38
TETPFGR	607	613	8,33	7,63	8,35	NaN
CHSAVDPAEYYK	614	625	7,51	6,78	7,93	NaN
CHSAVDPAEYYKR	614	626	7,85	NaN	7,96	NaN

PAEYYK	620	625	NaN	NaN	6,64	NaN
GVMLWGWR	656	663	NaN	6,95	7,40	NaN
CVPLAK	725	730	NaN	NaN	7,87	NaN
GLYLEAGDVVVR	737	748	9,69	8,35	9,01	8,26
GLYLEAGDVVVRQEER	737	752	8,20	7,08	7,81	NaN
LYLEAGDVVVR	738	748	7,15	NaN	NaN	NaN
LIGQSCTAPK	767	776	8,50	7,90	7,68	7,48
IGQSCTAPK	768	776	NaN	6,61	NaN	NaN
ATSKPR	788	793	NaN	7,61	NaN	NaN
TSKPR	789	793	NaN	7,87	NaN	NaN
GGCVVEK	825	831	7,19	7,33	NaN	NaN
ECPCVHNNDLYSSGAK	832	847	7,29	7,71	NaN	NaN
IKVDCNTCTCK	848	858	6,78	7,17	NaN	NaN
IKVDCNTCTCKR	848	859	NaN	6,98	NaN	NaN
VDCNTCTCKR	850	859	NaN	7,54	NaN	NaN
AIKIFMGR	932	939	8,01	7,93	NaN	7,26
IFMGR	935	939	8,17	8,15	NaN	NaN
TELKLEDK	940	947	8,34	7,99	6,77	6,98
TELKLEDKHR	940	949	8,22	8,58	6,68	7,08
VVIQR	950	954	8,53	8,63	7,17	7,20
VVIQRDEGHHVAYTTR	950	965	6,24	7,38	NaN	NaN
RDEGHHVAYTTR	954	965	NaN	8,68	8,36	NaN
DEGHHVAYTTR	955	965	8,33	8,25	7,14	7,01
TTVFIK	987	992	NaN	NaN	7,86	NaN
TTVFIKLAPSYK	987	998	7,82	7,56	NaN	NaN
LAPSYK	993	998	NaN	NaN	7,58	NaN
GTVCGLCGNFDHR	999	1011	7,83	8,47	7,72	NaN
SNNDFTTR	1012	1019	8,00	9,08	8,80	7,51
DHMVVSSELDFGNSWK	1020	1035	8,10	8,17	7,91	NaN
EAPTCPDVSTNPEPCSLNPHR	1036	1056	7,27	8,03	7,49	NaN
EAPTCPDVSTNPEPCSLNPHRR	1036	1057	7,06	7,76	7,32	NaN
SWAEK	1058	1062	6,78	7,17	6,75	NaN
SSVFSICHSK	1069	1078	8,36	8,33	8,16	NaN
VASYAQECTK	1108	1117	5,77	NaN	NaN	NaN
EGACVFWR	1118	1125	8,08	8,61	8,16	NaN
SFETCR	1154	1159	8,49	8,93	8,48	NaN
CPKDRPIYEEDLK	1180	1192	7,44	NaN	NaN	NaN
DRPIYEEDLK	1183	1192	8,02	8,42	8,37	NaN
DRPIYEEDLKK	1183	1193	8,71	8,99	8,68	NaN
KCVTADK	1193	1199	7,50	7,86	7,52	NaN
CGCYVEDTHYPPGASVPTEETCK	1200	1222	NaN	6,99	6,95	NaN
GAPEDIECR	1344	1352	NaN	6,75	NaN	NaN
VQCDVSVGFICK	1368	1379	7,76	8,35	7,70	NaN
VNCCWPMDK	1399	1407	NaN	6,77	NaN	NaN
CITSSEQK	1408	1415	6,98	6,84	6,13	NaN
FSVSGEGEGDATYGK	1457	1471	7,64	6,61	NaN	7,93
FSVSGEGEGDATYGKLTLK	1457	1475	7,50	NaN	NaN	NaN
SVSGEGEGDATYGK	1458	1471	NaN	NaN	NaN	6,10
LTLKFICTTGK	1472	1482	7,12	NaN	NaN	6,68
FICTTGK	1476	1482	7,29	NaN	NaN	7,46
QHDFFKSAMPEGYVQER	1510	1526	7,43	NaN	NaN	NaN
SAMPEGYVQER	1516	1526	7,96	7,04	NaN	8,65
TIFFK	1527	1531	7,51	NaN	NaN	8,09
TIFFKDDGNYK	1527	1537	8,36	7,27	NaN	8,81
TIFFKDDGNYKTR	1527	1539	7,24	NaN	NaN	7,38

AEVKFEGDTLVNR	1540	1552	8,51	7,21	NaN	8,93
FEGDTLVNR	1544	1552	8,12	NaN	NaN	NaN
IELKGIDFK	1553	1561	6,93	NaN	NaN	NaN
GIDFKEDGNILGHK	1557	1570	7,56	NaN	NaN	8,14
VNFKIR	1593	1598	7,71	NaN	NaN	7,95

Table S2 MUC2 peptide intensity

Peptide sequence	Start position	WT_1	WT_2	WT_3	WT_4	WT_5	WT_6	WT_7	WT_8	*Clca1*$^{-/-}$_1	*Clca1*$^{-/-}$_2	*Clca1*$^{-/-}$_3	*Clca1*$^{-/-}$_4	*Clca1*$^{-/-}$_5	*Clca1*$^{-/-}$_6	*Clca1*$^{-/-}$_7	*Clca1*$^{-/-}$_8
		Log10 normalized intensity															
NHVCSTWGDFHYK	31	-3.96	-2.53	-3.18	-2.82	-2.81	-2.44	-3.97	-2.59	-2.39	-2.93	-3.63	-3.21	-2.44	-3.75	-2.93	-2.75
TFDGDVYR	44	-1.77	-1.83	-1.73	-1.71	-1.68	-1.80	-1.72	-1.69	-1.78	-1.74	-1.69	-1.87	-1.77	-1.70	-1.70	-1.74
TFDGDVYRFPGLCDYNFASDCR	44	-2.16	-2.33	-3.30	-3.10	-2.71	-2.38	-2.72	-2.89	-2.47	-2.61	-3.49	-3.21	-2.36	-3.32	-3.05	-3.04
FPGLCDYNFASDCR	52	-2.21	-2.10	-1.97	-2.25	-1.94	-2.06	-2.00	-2.03	-2.11	-2.14	-2.21	-2.35	-2.13	-2.02	-2.05	-2.17
FPGLCDYNFASDCRDSYK	52	-3.40	-3.24	-3.57	-3.43	-3.30	-3.12	-3.32	-3.30	-3.13	-3.14	-3.59	-3.46	-3.28	-3.71	-3.40	
DSYKEFAVHLK	66	-2.48	-2.32	-2.48	-2.58	-2.63	-2.44	-2.47	-2.54	-2.51	-2.59	-2.67	-2.60	-2.67	-2.95	-2.26	-2.46
EFAVHLKR	70	-3.44	-3.69	-3.71	-3.46	-3.91	-3.54	-3.44	-3.39	-3.97	-3.73	-3.75	-3.62	-3.50	-4.02	-3.76	-3.56
GLGEAGGHSQIESILITIK	78	-3.36	-3.14	-3.82	-3.45	-3.44	-3.07	-3.34	-3.60	-3.37	-3.45		-3.61	-3.19	-3.75	-4.10	-3.57
DDTIYLTHK	97	-2.69	-2.70	-2.24	-2.24	-2.52	-2.60	-2.38	-3.39	-3.45	-2.58	-2.12	-2.35	-2.63	-2.40	-3.36	-2.24
LAVVNGAMVSTPHYSSGLLIEK	106	-1.85	-2.05	-2.09	-2.13	-2.14	-1.99	-1.91	-1.96	-2.07	-1.99	-2.08	-1.98	-2.16	-2.10	-2.12	-1.87
LAVVNGAMVSTPHYSSGLLIEKNDAYTK	106	-2.72	-2.55	-3.01		-4.05	-2.50	-2.63	-3.06	-2.62	-2.60	-3.25	-2.59	-2.60	-4.30	-3.06	-2.55
AGLSLMWNR	138	-2.92	-2.82	-3.25	-3.12	-2.79	-2.76	-2.80	-2.87	-2.64	-2.75	-3.33	-3.38	-3.01	-2.97	-3.36	-3.05
EDALMVELDSR	147	-2.64	-2.65	-2.42	-2.61	-2.42	-2.66	-2.63	-2.61	-2.88	-2.66	-2.66	-2.71	-2.81	-2.48	-2.60	-2.56
LLTSAAFEDCQTR	226	-1.54	-1.62	-1.70	-1.54	-1.54	-1.57	-1.53	-1.45	-1.70	-1.66	-1.54	-1.66	-1.81	-1.41	-1.41	-1.48
LLTSAAFEDCQTRVPVESYVR	226	-1.89	-1.79	-1.99	-1.93	-1.91	-1.90	-2.04	-1.97	-1.85	-1.75	-2.11	-1.97	-2.09	-2.35	-1.90	-2.19
VPVESYVR	239	-1.49	-1.62	-1.46	-1.45	-1.48	-1.55	-1.46	-1.42	-1.60	-1.50	-1.45	-1.58	-1.50	-1.42	-1.37	-1.44
GGACECSTLAEFSR	258	-1.78	-1.80	-1.82	-1.89	-1.73	-1.77	-1.84	-1.76	-1.80	-1.78	-1.77	-1.91	-1.97	-1.70	-1.73	-1.75
QCSHAGGRPENWR	272	-2.19	-2.01	-2.68	-2.37	-2.19	-2.11	-2.37	-2.18	-2.02	-2.20	-2.93	-2.83	-2.05	-2.81	-2.64	-2.58
TASLCPK	285	-1.52	-1.69	-1.49	-1.51	-1.62	-1.60	-1.51	-1.51	-1.66	-1.54	-1.48	-1.56	-1.50	-1.42		-1.49
LHGHLYMPGQEFTNDCEQCVCNAGR	353	-1.80	-2.72	-3.55	-2.29	-3.74	-1.89	-2.04	-1.92	-2.75	-2.69	-4.04	-2.14	-1.89	-3.65	-2.56	-2.10
DLPCPETCALEGGSHITTFDGK	382	-2.31	-2.39	-2.36	-2.52	-2.51	-2.33	-2.01	-2.40	-2.42	-2.41	-2.46	-2.39	-2.60	-2.43	-2.48	-2.31
DLPCPETCALEGGSHITTFDGKK	382	-2.48	-1.87	-1.99	-2.01	-1.95	-2.32	-2.02	-1.88	-2.08	-2.34	-2.13	-1.95	-2.74	-2.40	-1.91	-2.09
KFTFHGDCYYVLTK	404	-3.15	-3.68	-3.71	-3.41	-3.85	-2.99	-3.04	-3.10	-3.68	-3.83	-4.06	-3.16	-3.09	-4.35	-3.64	-3.16
FTFHGDCYYVLTK	405	-2.15	-2.35	-2.38	-2.45	-2.44	-2.17	-2.15	-2.23	-2.18	-2.46	-2.75	-2.19	-2.36	-2.73	-2.57	-2.17
TVVLLTDDK	444	-3.16	-3.28	-2.93	-3.02	-3.22	-3.25	-3.09	-3.07	-3.33	-3.19	-2.99	-3.14	-3.19	-3.03	-2.93	-3.16
TVVLLTDDKK	444	-1.53	-1.45	-1.65	-1.62	-1.65	-1.56	-1.58	-1.54	-1.74	-1.51	-1.42	-1.56	-1.59	-1.45	-1.38	-1.47
RSETPFAR	590	-2.35	-2.39	-2.50	-2.53	-2.39	-2.48	-2.47	-2.44	-2.42	-2.43	-2.55	-2.50	-2.38	-2.50	-2.56	-2.53
SETPFAR	591	-1.60	-1.63	-1.46	-1.51	-1.62	-1.68	-1.55	-1.52	-1.71	-1.63	-1.40	-1.56	-1.65	-1.47	-1.48	-1.49
CHLAVDPTEYYK	598	-2.31	-2.57	-2.48	-2.48	-2.51	-2.39	-2.49	-2.49	-2.50	-2.35	-2.41	-2.67	-2.61	-2.44	-2.25	-2.51
CHLAVDPTEYYKR	598	-1.58	-1.70	-1.70	-1.75	-1.91	-1.66	-1.66	-1.56	-1.51	-1.76	-1.95	-1.68	-1.29	-2.27	-1.63	-1.80
GVMLWGWR	640	-2.93	-2.22	-3.41	-2.92	-2.53	-2.61	-2.82	-2.95	-2.07	-2.36	-3.66	-5.03	-2.50	-3.42	-4.00	-4.37
SLSEGDSHCLK	677	-1.39	-1.63	-1.37	-1.37	-1.56	-1.49	-1.40	-1.42	-1.55	-1.37	-1.32	-1.43	-1.41	-1.34	-1.41	-1.36
GFAPVEGCGCPDHTFMDEK	688	-1.79	-1.95	-1.99	-1.91	-1.90	-1.92	-1.93	-1.78	-1.88	-1.96	-2.00	-2.05	-1.81	-1.94	-1.88	-1.89
GFAPVEGCGCPDHTFMDEKGR	688	-1.88	-1.81	-1.92	-2.32	-1.95	-1.87	-1.82	-1.79	-2.17	-1.84	-1.92	-1.83	-1.89	-2.14	-2.23	-1.81
CSCYHHGLYLEAGDVILR	715	-2.73	-3.01	-3.83	-3.47	-3.33	-2.75	-2.57	-3.10	-3.03	-3.55	-4.00	-3.32	-2.79	-4.05	-3.79	-2.89
CSCYHHGLYLEAGDVILRQEER	715	-2.78	-4.05	-4.49	-4.09	-3.96	-2.62	-2.78	-3.52	-3.49		-4.44	-3.26	-2.91	-4.06	-3.76	-3.26
LQCTQVK	744	-1.45	-1.38	-1.24	-1.27	-1.23	-1.38	-1.33	-1.32	-1.54	-1.35	-1.24	-1.33	-1.37	-1.17	-1.32	-1.33
LIGHTCQYPK	751	-1.82	-1.80	-1.99	-2.15	-1.76	-1.88	-1.99	-1.93	-1.73	-1.67	-1.80	-1.91	-1.70	-1.75	-1.76	-1.89
ILVDCNNLTALAVR	761	-3.40	-3.24	-3.29	-3.59	-2.74	-2.76	-2.87	-2.92	-3.50	-3.35	-3.55	-3.44	-3.36	-2.90	-3.08	-3.25
GGCVEEDK	809	-2.93	-2.83	-2.96	-2.75	-2.59	-3.05	-3.03	-2.92	-2.83	-3.10	-2.90	-3.10	-2.85	-2.70	-3.05	-2.99
GGCVEEDKCPCIHNK	809	-1.63	-1.66	-1.66	-1.68	-1.62	-1.74	-1.67	-1.62	-1.76	-1.71	-1.74	-1.72	-1.63	-1.76	-1.74	-1.80
CPCIHNK	817	-3.41	-3.55	-3.60	-3.40	-3.45	-3.57	-3.69	-3.50	-3.56	-3.59	-3.49	-3.69	-3.42	-3.41	-3.65	-3.64
DLYSSGESIK	824	-1.95	-2.07	-1.94	-1.89	-2.02	-2.06	-2.07	-1.99	-2.01	-2.08	-1.95	-2.03	-1.94	-1.91	-2.30	-2.02

YACHSTCSIYGSGHYITFDGK	852	-2.53	-2.77	-2.97	-2.68	-3.05	-2.50	-2.41	-2.56	-2.64	-2.71	-3.24	-2.59	-2.70	-3.29	-2.72	-2.32
IFIGGTELK	919	-1.39	-1.41	-1.24	-1.24	-1.42	-1.42	-1.38	-1.44	-1.36	-1.33	-1.20	-1.26	-1.70	-1.26	-1.25	-1.28
QLEEGHHVPYITR	937	-1.59	-1.54	-1.46	-1.48	-1.80	-1.69	-1.56	-1.67	-1.59	-1.58	-1.50	-1.51	-1.47	-1.95	-1.50	-1.54
TTIFIKLDPSYK	971	-3.81	-3.71	-4.08	-4.08	-4.04	-3.99	-4.05	-3.95	-3.61	-3.75	-4.13	-3.70	-3.86		-4.46	-4.18
LDPSYKGTVCGLCGNFDDQTK	977	-2.68	-2.52	-2.73	-2.62	-2.72	-2.75	-2.72	-2.85	-2.51	-2.63	-2.62	-2.50	-2.75	-2.67	-2.86	-2.67
GTVCGLCGNFDDQTK	983	-1.97	-2.04	-2.05	-1.98	-1.99	-2.05	-2.19	-2.08	-2.08	-2.09	-2.05	-2.16	-2.23	-2.00	-2.06	-2.24
GTVCGLCGNFDDQTKNDFTTR	983	-1.86	-2.04	-2.06	-1.82	-2.11	-2.04	-1.92	-1.92	-1.78	-1.88	-1.96	-1.83	-2.01	-2.14	-2.01	-1.85
DHMVVTSELDFGNSWK	1004	-2.57	-2.35	-2.79	-2.51	-2.52	-2.52	-2.69	-2.57	-2.12	-2.46	-2.99	-4.35	-2.53	-3.12	-3.64	-2.88
EASTCPDVSHNPDPCSLNPHR	1020	-2.51	-2.49	-2.74	-2.70	-2.71	-2.53	-2.69	-2.52	-2.64	-2.54	-2.73	-2.64	-2.61	-2.65	-2.55	-2.63
EASTCPDVSHNPDPCSLNPHRR	1020	-1.83	-2.32	-2.33	-1.75	-2.70	-1.81	-1.81	-1.80	-2.56	-2.67	-2.88	-1.81	-1.95	-3.40	-2.16	-1.85
AEACVFWR	1102	-2.35	-2.26	-2.41	-2.44	-2.12	-2.19	-2.28	-2.29	-1.81	-2.08	-2.64	-2.84	-2.43	-2.34	-2.52	-2.47
CPEDRPIYDEDLK	1164	-2.31	-2.41	-2.23	-2.27	-2.39	-2.46	-2.28	-2.19	-2.43	-2.47	-2.34	-2.47	-2.45	-2.22	-2.33	-2.41
CGCYIEDTR	1184	-3.13	-3.14	-3.17	-3.17	-3.10	-3.35	-3.30	-3.16	-3.25	-3.28	-3.10	-3.37	-3.27	-2.99	-3.14	-3.35
CGCYIEDTRYPPGGSVPTDEICK	1184	-2.15	-2.07	-2.12	-2.27	-2.11	-2.21	-2.16	-2.08	-2.22	-2.13	-2.21	-2.20	-2.10	-2.08	-1.86	-2.25
YPPGGSVPTDEICK	1193	-3.36	-3.35	-2.82	-3.19	-3.15	-3.40	-3.28	-3.18	-3.45	-3.52	-3.23	-3.43	-3.55	-2.99	-3.07	-3.36
SCTCTNTSK	1207	-4.41	-4.68	-4.60	-4.58	-3.62	-4.12	-4.15	-4.01	-4.66	-4.54		-4.69	-4.23	-4.34	-4.45	-4.39
IECHPDEGK	1216	-1.35	-1.34	-1.34	-1.34	-1.21	-1.44	-1.41	-1.36	-1.32	-1.36	-1.29	-1.42	-1.36	-1.20	-1.41	-1.40
YHPTKENGGDR	1306	-3.54	-3.14	-3.54	-3.29	-3.29	-3.57	-3.62	-3.57	-3.11	-3.80	-3.65	-3.49	-3.50	-3.82	-3.47	-3.39
ENGGDRETFTHVCSAPEDIECR	1311	-1.43	-1.45	-1.30	-1.47	-1.60	-1.44	-1.45	-1.62	-1.55	-1.52	-1.60	-1.45	-1.51	-1.87	-1.77	-1.63
ETFTHVCSAPEDIECR	1317	-2.29	-2.46	-2.38	-2.22	-2.28	-2.37	-2.37	-2.40	-2.54	-2.34	-2.30	-2.41	-2.36	-2.27	-2.74	-2.37
LSWEELGQK	1339	-1.77	-1.74	-2.02	-2.09	-1.87	-1.82	-1.82	-1.90	-1.41	-1.70	-2.14	-2.24	-2.06	-2.03	-2.07	-2.08
WTGWLDSGKPTYDIK	1521	-2.62	-2.47	-3.43	-3.00	-2.74	-2.54	-2.96	-2.89	-2.14	-2.41	-3.60	-3.28	-2.65	-3.66	-3.28	-3.23
SGDFELIK	1536	-1.29	-1.15	-1.40	-1.14	-1.36	-1.38	-1.29	-1.31	-1.24	-1.30	-1.15	-1.14	-1.45	-1.20	-1.20	-1.23
GVCEPHWEVQNISCR	1544	-3.54	-3.47	-4.26	-3.84	-3.25	-3.09	-3.27	-3.31	-3.56	-3.59	-3.98	-3.98	-3.62	-3.52	-3.36	-3.62
AVMHSNIPLDQLGQIVVCNK	1559	-1.92	-1.21	-2.94	-1.48	-1.37	-1.25	-2.27	-2.60	-1.25	-1.80	-1.63	-2.56	-1.78	-1.77	-1.42	-2.79
EVGLVCK	1579	-1.86	-1.94	-1.73	-1.72	-1.84	-1.84	-1.90	-1.82	-1.98	-1.92	-1.67	-1.80	-1.83	-1.67	-1.75	-1.73
EVGLVCKNEDQEIGGIIPMR	1579	-1.68	-1.44	-1.82	-1.94	-1.87	-1.81	-1.64	-1.82	-1.66	-1.74	-1.84	-1.68	-1.93	-1.97	-1.68	-1.86
NEDQEIGGIIPMR	1586	-1.94	-1.96	-1.72	-1.85	-2.02	-2.03	-2.03	-1.96	-1.97	-2.01	-1.87	-1.91	-2.10	-1.80	-1.69	-1.88
STPSTPQSTSPK	1912	-3.09	-2.54	-3.35	-3.18	-3.20	-3.00	-3.29	-3.23	-3.75	-3.12	-3.07	-3.05	-2.99	-2.38	-2.73	-3.54
STLSTPTK	1924	-3.01	-2.61	-3.29	-3.16	-3.26	-2.93	-3.20	-3.16	-3.74	-3.07	-3.02	-2.97	-2.90	-2.34	-2.69	-3.37
TTPYGCPDFDPPR	1932	-3.65	-3.17	-4.18	-4.03	-3.82	-3.54	-3.94	-3.89	-4.23	-3.78	-3.78	-3.83	-3.77	-2.97	-3.31	-4.20
CDPPPMPTCANGLKPVR	1973	-3.18	-1.92	-2.27	-2.57	-2.08	-2.15	-2.38	-3.57	-1.94	-3.42	-2.37	-2.54	-2.34	-2.05	-2.26	-2.13
TLIVRHETQEVQIK	2063	-3.00	-3.21	-2.84	-3.25	-2.99	-2.79	-3.04	-3.17	-3.08	-3.39	-3.03	-2.86	-2.86	-3.34	-3.19	-3.09
HETQEVQIK	2068	-1.76	-1.87	-1.59	-1.70	-1.72	-1.64	-1.63	-1.62	-1.84	-1.68	-1.62	-1.55	-1.68	-1.53	-1.58	-1.60
MMPIEVEVQVNK	2080	-2.16	-2.24	-2.05	-2.09	-2.08	-2.00	-2.02	-1.97	-2.10	-2.16	-2.06	-1.95	-2.10	-2.02	-2.00	-2.09
QLVALPYK	2092	-2.67	-2.61	-2.29	-2.57	-2.50	-2.48	-2.48	-2.53	-2.71	-2.56	-2.39	-2.43	-2.70	-2.43	-2.55	-2.55
QLVALPYKK	2092	-2.05	-2.04	-1.47	-2.02	-1.67	-1.85	-1.91	-2.03	-2.06	-1.83	-1.82	-1.86	-1.89	-1.85	-2.20	-2.02
ISYNGLSFSIR	2123	-2.09	-1.94	-1.97	-1.91	-1.73	-1.77	-1.87	-1.85	-1.90	-1.99	-2.12	-1.84	-2.07	-1.92	-2.28	-2.01
EGVCIDWR	2265	-3.68	-3.39	-3.96	-3.63	-3.58	-3.29	-3.46	-3.44	-3.48	-3.48	-3.97	-4.21	-3.71	-3.41	-4.23	-3.77
FAPGYDVCVK	2324	-2.01	-2.02	-1.96	-2.05	-1.79	-1.87	-1.99	-1.94	-2.07	-1.95	-1.96	-1.94	-2.16	-1.75	-1.88	-2.00
TCGCVGPDNVPR	2334	-2.33	-2.48	-2.39	-2.36	-2.15	-2.34	-2.27	-2.20	-2.65	-2.42	-2.35	-2.31	-2.45	-2.12	-2.15	-2.33
EFGEHFEFDCK	2346	-3.13	-3.26	-3.00	-3.28	-3.03	-3.11	-3.01	-2.98	-3.27	-3.32	-3.34	-3.21	-3.18	-3.34	-3.07	-3.06
EFGEHFEFDCKDCVCR	2346	-2.86	-3.05	-3.13	-3.13	-2.90	-2.63	-2.71	-2.73	-3.07	-3.23	-3.56	-2.97	-2.80	-3.44	-2.92	-2.88
EGGSGIVCQPK	2362	-2.14	-2.26	-2.13	-2.28	-1.91	-2.02	-2.18	-2.02	-2.26	-2.13	-2.17	-2.16	-2.09	-1.96	-2.09	-2.23
EGGSGIVCQPKK	2362	-3.06	-2.81	-2.79	-2.94	-2.55	-2.77	-2.90	-2.81	-2.95	-2.80	-2.80	-2.80	-2.81	-2.76	-2.92	-2.97
AERPTCLLGFEVK	2414	-2.52	-2.60	-2.62	-2.79	-2.55	-2.58	-2.57	-2.42	-2.72	-2.53	-2.74	-2.63	-2.59	-2.84	-2.18	-2.70
PTCLLGFEVK	2417	-4.01	-4.10	-3.95	-3.94	-3.74	-3.73	-3.64	-3.87		-3.87	-4.18	-3.92	-4.03	-3.77	-4.08	-3.98
CCPVYSCVPK	2434	-2.33	-2.33	-2.05	-2.39	-2.07	-2.18	-2.21	-2.11	-2.18	-2.12	-2.12	-2.36	-2.11	-2.08	-2.08	-2.37

GVCVHQNAEYQPGSPVYSNK	2444	-1.96	-2.09	-1.77	-2.00	-1.85	-1.91	-1.89	-1.82	-1.96	-1.86	-1.86	-1.83	-1.85	-1.88	-1.64	-1.86
KCQQTHCIIEGPK	2509	-3.48	-3.50	-3.45	-3.49	-3.33	-3.43	-3.57	-3.26	-4.12	-3.68	-3.72	-3.39	-3.35	-4.09	-3.54	-4.67
CQQTHCIIEGPK	2510	-2.43	-2.38	-2.24	-2.51	-2.23	-2.16	-2.35	-2.24	-2.37	-2.10	-2.25	-2.34	-2.23	-2.14	-2.16	-2.39
QQYIILKPGEIHK	2522	-2.15	-2.44	-2.38	-2.48	-2.33	-2.16	-2.22	-2.25	-2.30	-2.35	-2.53	-2.31	-2.12	-2.73	-2.18	-2.22
CTFFSCMK	2540	-2.50	-2.52	-2.30	-2.61	-2.23	-2.29	-2.38	-2.46	-2.49	-2.35	-2.37	-2.54	-2.44	-2.31	-2.56	-2.57
VPCSAVSVMK	2593	-2.21	-2.34	-2.17	-2.32	-2.06	-2.11	-2.17	-2.11	-2.20	-2.12	-2.14	-2.18	-2.13	-2.03	-2.04	-2.24
EISYNGCTK	2603	-2.71	-2.65	-2.85	-3.02	-2.49	-2.67	-2.78	-2.67	-2.64	-2.60	-2.69	-2.84	-2.55	-2.49	-2.65	-2.77
CSCCKEEK	2639	-3.21	-3.22	-2.85	-3.46	-2.73	-3.32	-3.27	-3.16	-3.27	-3.05	-2.88	-3.41	-3.35	-2.69	-2.79	-3.41

References

1. Dong X, Leksa NC, Chhabra ES, et al. The von Willebrand factor D′D3 assembly and structural principles for factor VIII binding and concatemer biogenesis. *Blood*. 2019;133(14):1523-1533.
